# Natural Bioactive Cinnamoyltyramine Alkylamides and Co-Metabolites

**DOI:** 10.3390/biom11121765

**Published:** 2021-11-25

**Authors:** Antonio Evidente, Marco Masi

**Affiliations:** Department of Chemical Sciences, University of Naples Federico II, Complesso Universitario Monte Sant’Angelo, Via Cintia 4, 80126 Naples, Italy; marco.masi@unina.it

**Keywords:** alkylamide, cinnamoyltyramine, plant sources, different carbon skeleton, biological activity

## Abstract

Natural products are a vital source for agriculture, medicine, cosmetic and other fields. Among them alkylamides are a broad and expanding group found in at least 33 plant families. Frequently, they possess a simple carbon skeleton architecture but show broad structural variability and important properties such as immunomodulatory, antimicrobial, antiviral, larvicidal, insecticidal and antioxidant properties, amongst others. Despite to these several and promising biological activities, up to today, only two reviews have been published on natural alkylamides. One focuses on their potential pharmacology application and their distribution in the plant kingdom and the other one on the bioactive alkylamides specifically found in *Annona* spp. The present review is focused on the plant bioactive cinnamoyltyramine alkylamides, which are subject of several works reported in the literature. Furthermore, the co-metabolites isolated from the same natural sources and their biological activities are also reported.

## 1. Introduction

Alkylamides are a broad and expanding group of bioactive natural compounds grouped at least in 33 plant families as Aristolochiaceae, Asteraceae, Brassicaceae, Convolvulaceae, Euphorbiaceae, Menispermaceae, Piperaceae, Poaceae, Rutaceae and Solanaceae [1]. Many of these species were used in folk medicine for the broad spectra of biological activities as immunomodulatory, antimicrobial, antiviral, larvicidal, insecticidal, diuretic, analgesic, cannabimimetic and antioxidant activities. They are also involved in the antibiotic’s potentiation, the prostaglandin biosynthesis inhibition, RNA synthesis and the arachidonic acid metabolism. Alkylamides possess a broad range of pharmacological effects [2] and thus their potential application in the pharmaceutical, cosmetic and food industries could be planned. Alkylamides are found in different organs of the plants such as roots, leaves, stems, fruits, flowers, seeds and tubers. Alkylamides were also formulated as plant growth regulators, which affect the growth, roots development and inducing of plant biomass production [3]. 

**Figure 1:**
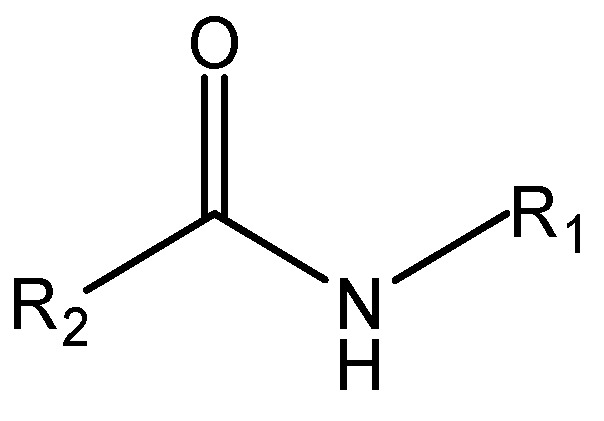
General structure of an alkylamide.

Natural alkylamides are constituted by an aliphatic, cyclic or aromatic amine residue (R_1_), and a C8 to C18 saturated or unsaturated chain acid, which can also be aromatic (R_2_). The structural formula representing all the alkylamides is reported in Figure 1.

The nature of the acid and the amine residues are characteristic of each plant family and species. They are also classified as protoalkaloid or pseudoalkaloid compounds and represent a group of lipidic compounds structurally related to animal endocannabinoids and are strongly active metabolites in the central nervous system. Some previous reviews reported the chemistry and the biological activity of alkylamides, and although they cover a broad range of literature, they are organized differently. One was organized accordingly to the family of the plant source [4], and another one reported the chemistry and the detailed description of their biological activities [5].

The present review is focused on the cinnamoyltyramine subgroup of the alkylamides, reporting their biosynthesis, chemical structures, biological activities, hemisynthetic derivatives and structure activity studies. Furthermore, the co-metabolites isolated from the same natural sources and their biological activity are also described.

## 2. Biosynthesis of *N*-*trans*-Cinnamoyltyramine

**Scheme 1:**
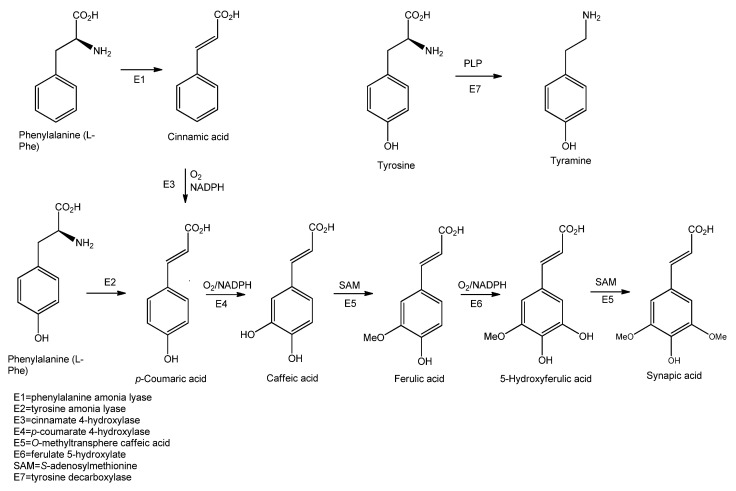
Biosynthesis of cynnamic acids and tyramine from phenylalanine and tyrosine, respectively.

The biosynthesis of *N*-*trans*-cinnamoyltyramine (**1**) in plants could occur in several steps. The biosynthetic pathway starts from *trans*-cinnamic acid and tyramine, which were, respectively, generated from phenylalanine (L-Phe), as were the other cinnamic acids (i.e., *p*-coumaric, caffeic, ferulic, 5-hydroxyferulic and sinapic acids) and tyrosine (Tyr), as reported in Scheme 1. 

**Scheme 2:**
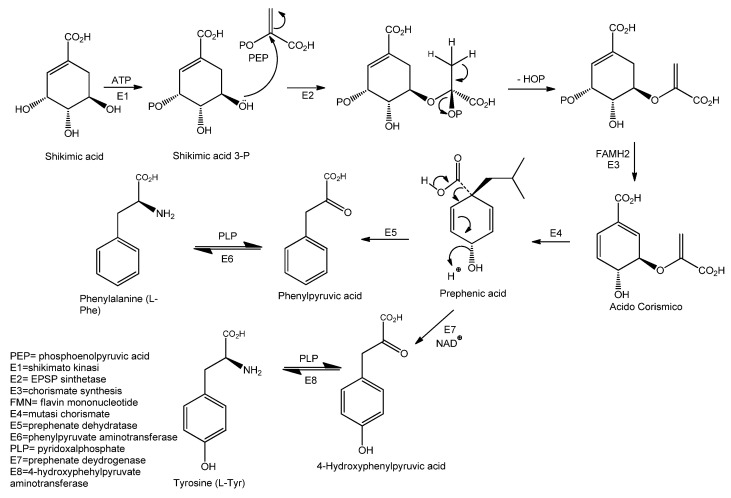
Biosynthesis of phenylalanine and tyrosine (Phe and Tyr) according to shikimic acid pathway.

Both aromatic amino acids (Phe and Tyr) were synthesized from prefenic acid, which was, in turn, generate from shikimic acid according to the shikimate pathway [6,7] reported in Scheme 2.

**Scheme 3:**
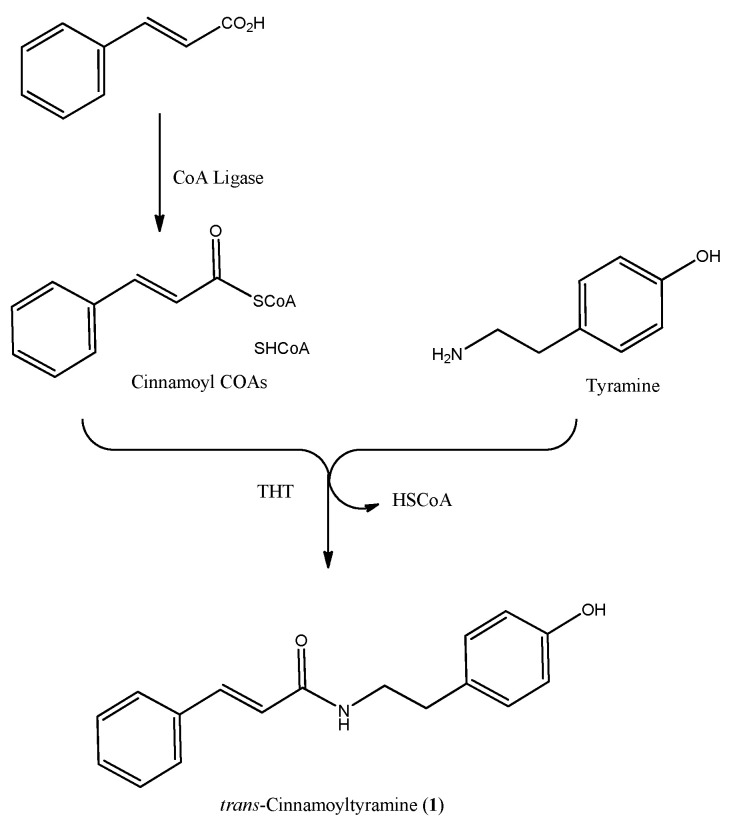
Biosinthesis of *N*-*trans*-cinnamoyltyramine (**1**) by conjugation of cinnamoylCoA and tyramine.

In particular, Phe was converted by phenylalanine ammonia-lyase into cinnamic acid according to [7,8], and tyramine was synthesized by decarboxylation of tyrosine as reported in Scheme 1 [7,9]. As reported in Scheme 3, cinnamic acid was converted by COA ligase into the corresponding activate form [10]. The final step provides the conjugation of cinnamoylCoA and tyramine catalyzed by the tyramine *n*-hydroxycinnamoyl transferase (THT): this enzyme is not specific to cinnamoylCoA and tyramine, but also catalyzes the conjugation of tyramine with the other CoA-activated cinnamic acids cited above [11,12].

## 3. Structure and Biological Activity of Cinnamoyltyramine Alkylamides and of Co-Metabolites Isolated from the Same Natural Sources

This section describes the structure and stereostructure determination of both *E*- and *Z*-diastereomers of *p*-coumaroyl-, caffeoyl-, feruloyl-, 5-hydroxyferuloyl- and sinapoyl-tyramine alkylamides, including a few uncommon analogues and their biological activities. Their promising practical applications are also described. Furthermore, chemical and biological aspects of the co-metabolites isolated from the same sources are described.

**Figure 2:**
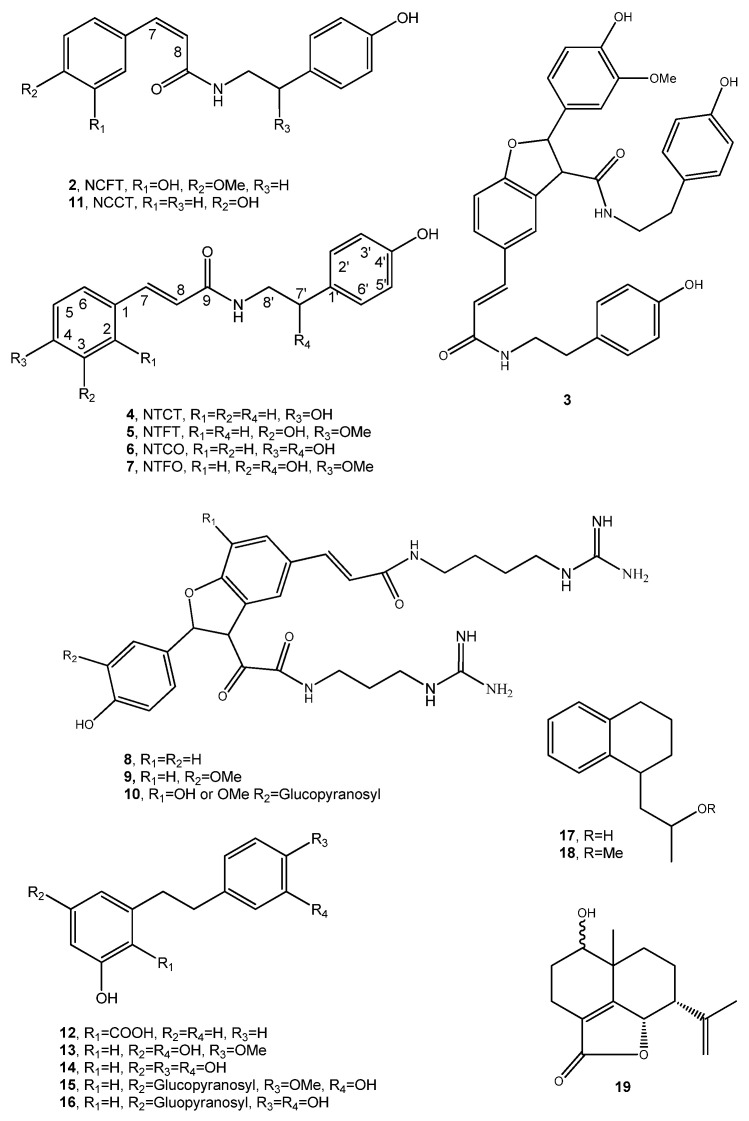
The structures of compounds **2**–**19**.

*N*-*cis*-feruloyltyramine (NCFT) and grossamide (**2** and **3**, Figure 2), two previously undescribed phenolic amides, were isolated from the roots of bell pepper (*Capsicum annuum* var. *grossum*, Solanaceae) together with *p*-aminobenzaldehyde and other alkylamides as *N*-*trans*-*p*-coumaroyltyramine (NTCT, also called prapazine), *N*-*trans*-feruloyltyramine (NTFT), *N*-*trans*-*p*-coumaroyloctopamine (NTCO) and *N*-*trans*-feruloyloctopamine (NTFO) (**4**–**7**, Figure 2) [13,14]. These latter compounds were previously isolated from the roots of eggplant (*Solanum melongena* L., Solanacee) [15]. The structure of grossamide was confirmed by its synthesis starting from *N*-*trans*-feruloyltyramine by an oxidative radical coupling. It is classified into a group of lignin accordingly McCredie et al. (1969) [16], who suggested to include in the lignin group all low molecular weight natural compounds that were generated from the oxidative coupling of *p*-hydroxyphenylpropene [13].

Very few studies have been reported on oxidative coupling products possessing amide groups. Among them there are hordatines A, B and M (**8**–**10**, Figure 2) found as antifungal factors in barley (*Hordeum vulgare*, Graminacee) [17]. Hordatin M is a mixture of glucosides of hordatins A and B. Hordatins belong to polyammide, whose biosynthesis started from *p*-hydrocynnamic acid CoA and agamatine obtained from decarboxylation of arginine. Then agmatinecoumaroyl transerase (ACT) catalyzes agamatine conjugates from coumaroyl- or feruloyl-CoA to give the corresponding *p*-hydrocinnamoylagamantine amide. The latter generate the dimeric hordatines by peroxidase oxidation [18]. Hordatines showed significant antifungal activities [19,20] and are biosynthesized as pro-defense compounds in barley seedlings or are accumulated in plants after a pathogen attack [21,22]. 

NTCT and *N*-*cis*-*p*-cumaroyltyramine (NCCT, **11**, Figure 2), lunularic acid (**12**, Figure 2) and *p*-coumaric acid were isolated from bulbs of *Allium chinense* (Amaryllidaceae), which is used in Chinese folk medicine [23]. They are well known as inhibitors of prostaglandin (PG) and thromboxane synthetases. Rhapontigenin, piceatannol, rhaponticin and piceatannol glucoside (**13**–**16**, Figure 2) are stilbene derivatives structurally related to lunularic acid and obatined from rhubarb (*Rheum rhabarbarum*, Polygonaceae) [24]. They were tested among other analogues to evaluate their effect on prostaglandin synthetase, using platelet-rich plasma (PRP) obtained from blood collected from the main leg artery of a male albino rabbit. Rhapontigenin showed the most potent inhibition on PG-ase and strongly inhibited platelet aggregation induced by arachidonic acid and collagen. Platelet aggregation was demonstrated in in vivo studies. The balance between thromboxane (TX) A_2_ and prostaglandin (PG) I_2_ (prostacyclin) plays a very important role in the regulation of blood flow. In fact, an excessive platelet aggregation is responsible to co-cause thrombosis and arteriosclerosis. Consequently, the inhibitory effect against PG or TX biosynthesis showed by the stilbene metabolites isolated from *A*. *chinese* could have an important therapeutic potential [23].

NTFT was successively isolated together with new alkaloids, papracinine and paprazine, and six already known ones, fumaritine *N*-oxide, parfumine, lastourvilline, fumariflorine and *N*-methyl corydaldine from the aerial parts of *Fumaria indica* (Papaveraceae), which is diffused in Europe, Central Asia and Africa. However, no activity was reported [25]. In the same year, but from the bark ethanolic extract of *Asimina triloba* L. (Annonaceae), NTCT and NTFT were isolated by a bio-guided fractionation testing brine shrimp lethality, together with a previously undescribed cytotoxic compound named acetogenin, and some known compounds such as asimicin, bullatacin, bullatacinone and (+)-syringaresinol. *A. triloba* L., an Annonaceae, commonly known as the pawpaw tree, which is native to the United States and spread in Europe, has been prized for its delicious, custard-like fruit. Trilobacin is a diastereomer of asimicin and both compounds showed potent and selective cytotoxicities in the NCI human tumor cell line screen [26].

NTCT was isolated from the stem bark extracts of *Isolona maitlandii* (Annonaceae), together with hexalobine-type, aporphinoids, amides and sterols. The leaf extract contained only hexalobines including ent-hexalobine C and five previously undescribed hexalobines. Any biological activity was reported [27]. 

**Figure 3:**
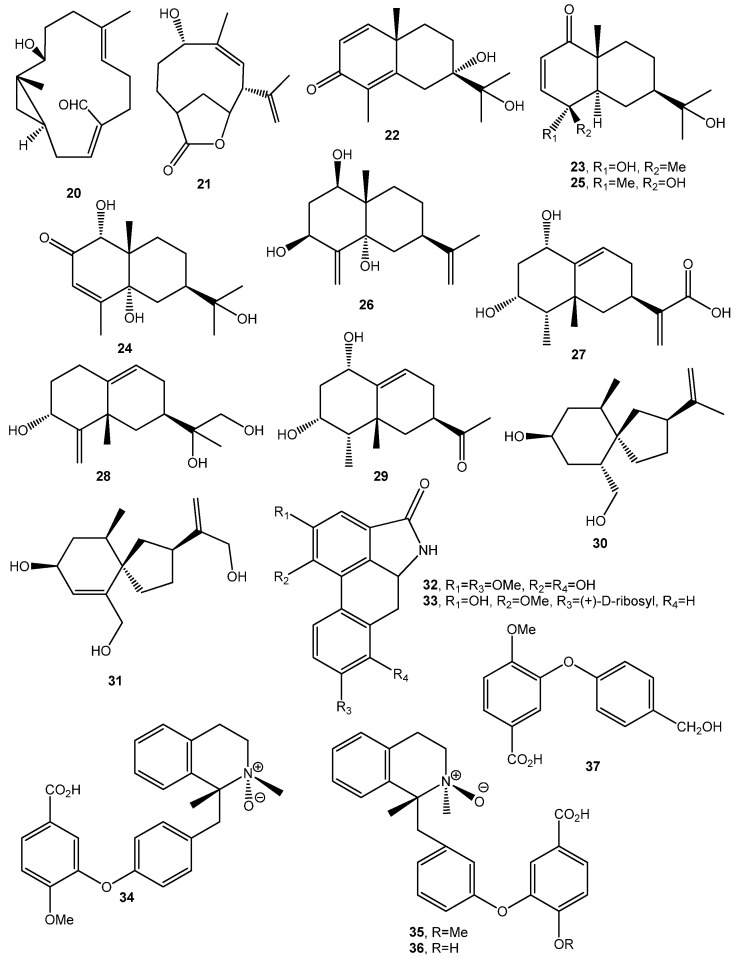
The structure of compounds **20**–**37**.

NTCT and NCCT were isolated also from *Aristolochia mollissima* belong to Aristolochiaceae. *Aristolochia* is a genus constituted by ca. 400 species that are widely distributed from the tropics to temperate regions. The roots and fruits of *A. mollissima* are used in Chinese folk medicine as analgesic, anticancer, antimalarial and anti-inflammatory agents, and also for the treatment of stomachache, abdominal pain and rheumatism. New sesquiterpenes, named mandolins S, R, U (**17**–**19**, Figure 2), W and X (**20** and **21**, Figure 3), together with 38 already known compounds belonging to different groups of natural compounds, were isolated from this plant [28].

NCFT, NTCT and NTFT were again isolated together with NCCT (**11**) and the already known lariciresinol, 13-hydroxycapsidiol, lubiminol and drummondol from red pepper (*Capsicum annuum*) (Solanaceae). However, the main metabolite isolated from *C. annnum* was capsaicin, a compound known to be responsible of pungent activity, and the plant was studied for its components, dietary effects and analgesic antioxidant activity [29,30]. Furthermore, 10 previously undescribed co-metabolites (eight bicyclic and two spiranic sesquiterpenes) were isolated from the same plant and named canusesnol A–J (**22**–**31**, Figure 3). The sesquiterpenes and the known compounds showed scant cytotoxic and anti-HIV activity [31].

NTFT and NTCT were isolated together with an azanthracene alkaloid, characterized as 1-aza-9,10-dimethoxy-4-methyl-2-oxo-1,2-dihydroanthracene and named kalasinamide, from the stems of *Polyalthia suberosa* (Annonaceae), which is a shrubby tree spread between southeast Asia and south China [32]. From the organic extract of its stems and leaves collected in China, a triterpene was previously isolated, named suberosol, with anti-HIV activity [33]. Successively, from the same plant together with NTFT and NTCT, two undescribed 2-substituted furans, 1-(2-furyl)pentacosa-16,18-diyne and 23-(2-furyl)tricosa-5,7-diynoic acid [34], were also isolated. As NTCT was isolated in limited amount not sufficient to investigate its biological activity, its synthesis was realized in one step starting from coumaric acid and tyramine with a final yield about of 55%. It showed suppression of growth of human tumor cells, such as U937 and Jurkat cells, which appeared associated with an increased percentage of cells in the S phase of the cell cycle progression. Furthermore, NTCT was able to inhibit the protein tyrosine kinases including epidermal growth factor receptor (EGFR) [35].

NTCT was isolated from twigs of *Celtis chinensis,* which was used in folk medicine in Korea, Japan and China to treat lumbago, irregular menstruation and gastric diseases [36]. Furthermore, NTCT inhibited acetylcholinesterase (ACHE), a well-known enzyme that plays an important role in Alzheimers disease [37].

The same four alkymides, NCFT, NTCT, NTFT and NCCT, were again isolated together with other already known compounds, belonging to different classes of naturally occurring compounds, from the root and stem of *Aristolochia elegans* [38]. *A. elegans* belong to the genus Aristolochia (Aristolochiaceae), and the alcoholic extracts of some species were investigated for their uterus contraction stimulating [39], antimitotic and antiviral properties [40]. *A. elegans* also produced previously undescribed compounds characterized as two aristolactams, aristolactam E and aristolactam-AIIIa-6-*O*-β-d-glucoside (**32** and **33**, Figure 3), three benzoyl benzyltetrahydroisoquinoline ether *N*-oxide alkaloids, aristoquinolines A–C (**34**–**36**, Figure 3), as well as a biphenyl ether, aristogin F (**37**, Figure 3). All the metabolites were tested to evaluate their potential antioxidative and antityrosinase properties, but neither the four alkylamines or the new metabolites showed activity [38].

**Figure 4:**
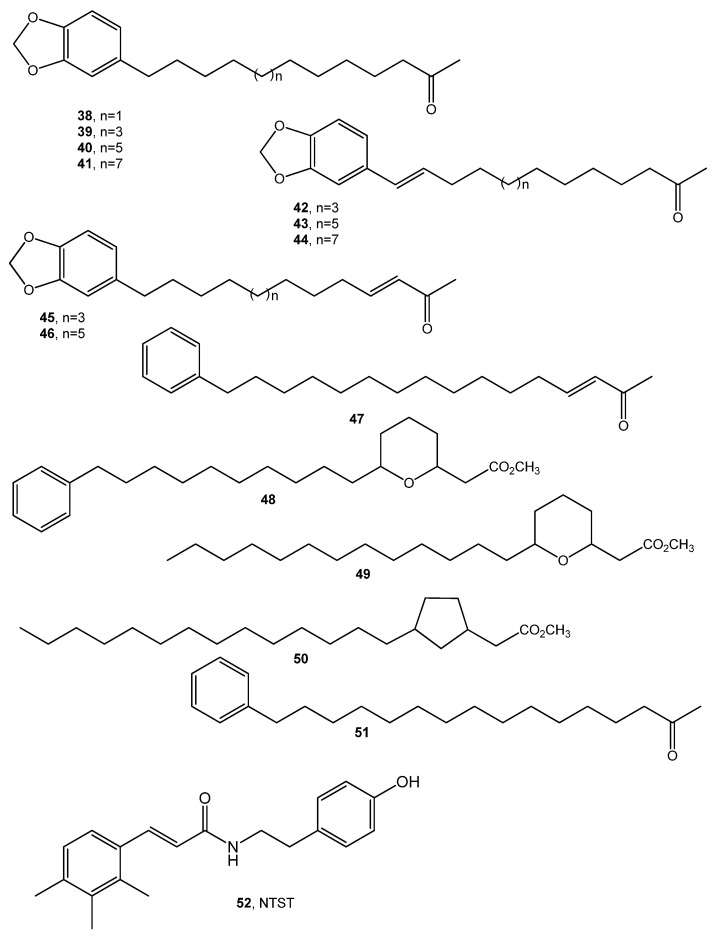
The structures of compounds **38**–**52**.

NTCT and NTFT were isolated from the organic extract of leaves and stems of *Piper sanctum* (Piperaceae) collected in Mexico together with nine monosubstituted 8-benzo[*d*] [1,3]dioxole (**38**–**46**, Figure 4), three monosubstituted alkylbenzene, a 2,6-disubstituted tetrahydropyranone and a 2,5-disubstituted tetrahydrofuranone. From the same extract were also isolated *p*-eugenol, methyleugenol, *Z*-piperolide, demethoxyyangonin 5,6-dehydro-7,8-dihydromethysticin, cepharanone B, piperolactam A, cepharadione B and safrol, which was the major component of the oily extract. Compounds **38**, **39**, **43,** demethoxyyangonin, 5,6-dehydro-7,8-dihydromethysticin, cepharanone B, piperolactam A and NTCT inhibited the growth of *Mycobacterium tuberculosis* when tested by the Microplate Alamar Blue Assay (MABA), with MIC values ranging from 4 to 64 μg/mL [41].

*N*-*trans*-sinapoyltyramine (NTST, **52**, Figure 4), NCFT, NTFT and NTCT were isolated together with 23 known compounds from the bark stems of *Polyalthia longifolia* var. *pendula* [40], while NTFT and NTCT were also isolated from *Sparattanthelium tupiniquinorum* (Hernandiaceae) collected in Brazil [42,43].

**Figure 5:**
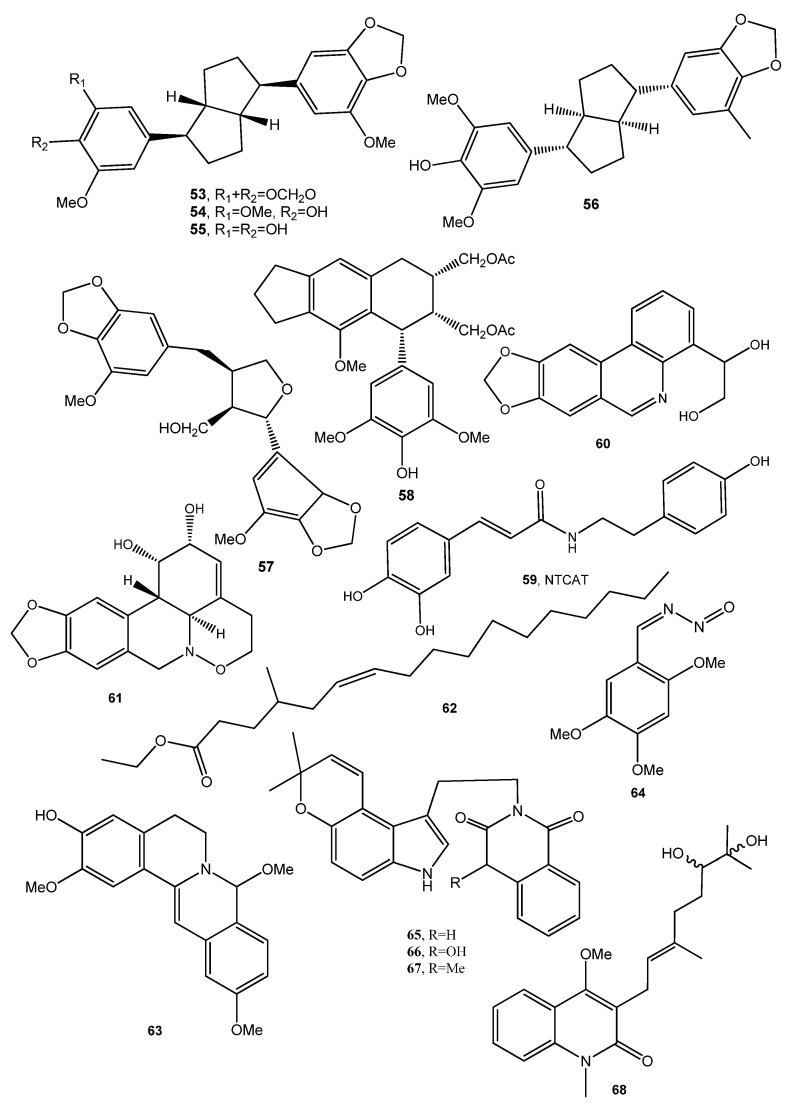
The structures of compounds **53**–**68**.

NTCT, NCCT and NTFT were isolated together with six previously undescribed lignans (**53**–**58**, Figure 5), and 11 other types of known compounds from *Peperomia duclouxii* (Piperaceae), which is a plant used in folk medicine as an anticancer agent in mainland China. When these compounds were tested in cytotoxic and MDR (multidrug resistance) reversal cell activity assays, only compound **55** inhibited the growth of VA-13 and HepG2 cancer cells, with IC_50_ values of 5.3 and 13.2 μg/mL, respetively. Compound **55** also showed potent effects on calcein accumulation in MDR 2780AD cells than verapamil, which was used as a positive control. Compound **58** exhibited anti-inflammatory activity using an ICAM-1 assay (induction of the intercellular adhesion molecule-1) and stimulated IL-1α (Interleukin 1 alpha) and TNF-α (tumor necrosis factor alpha) with IC_50_ values of 107 and 13.4 μM, respectively, and without cytotoxicity against A549 cells [44].

NTCT was isolated together with cannabisin G and (±)-lyoniresinol from the organic extract of the root bark of *Berberis vulgaris* L. (Berberidaceae). Different parts of this species were used for the treatment of diarrhea, gallbladder and liver dysfunctions, leishmaniasis, malaria, stomach problems and urinary tract diseases [45]. Cannabisin G and (±)-lyoniresinol, using a hydroxyl radical scavenging assay, exhibited antioxidant activity, while cannabisin G showed cytoprotective activity in cultured MCF-7 cells [46].

NTCT and *N*-*trans*-caffeoyltyramine (NTCAT, **59**, Figure 5) were isolated together with two new alkaloids, named asiaticumines A and B (**60** and **61**, Figure 5), and 18 other known compounds from the CHCl_3_ and EtOAc extracts of *Crinum asiaticum* var. sinicum Baker bulbs. This plant belongs to a well-known subgroup of Amaryllidceae, which synthesize alkaloids with several biological activities [47,48,49,50,51]. This species was used in traditional Chinese medicine for the treatment of abscesses, aching joints and sores. The already known metabolites were identified as the alkaloids crinumaquine, lycorine, hippacine, ungeremine, 11-*O*-methylcrinamine, 3-*O*-acetylhamayne, crinamine, criwelline and 4-hydroxystyryolamine. The other metabolites were identified as follows: 4-aminobenzaldehyde; the three flavonoids, such as (2*S*)-3′,7-dihydroxy-4′-methoxyflavan, 7-hydroxyflavanone and 4′,7-dihydroxyflavone; and the five phenolic compounds, such as *trans*-caffeic acid, 4-coumaric acid, 4-hydroxybenzonic acid, ethyl 4-hydroxybenzoate and 2-(3,4-dihydroxyphenyl)-1,3-benzodioxole-5-carboxaldehyde. When the alkaloids **60** and **61** and the other alkaloids were assayed for their cytotoxic activities against the human tumor cell lines A549, LOVO, HL-60 and 6T-CEM, only crinumaquine, lycorine, ungeremine, 11-*O*-methylcrinamine, 3-*O*-acetylhamayne and crinamine showed inhibition against one or more of the tested cell lines [52].

NTCT was isolated together with two previously undescribed compounds, namely 4-methyl-heptadec-6-enoic acid ethyl ester and 3-hydroxy-2,9,11-trimethoxy-5,6-dihydro isoquino[3,2-a]isoquinolinylium (**62** and **63**, Figure 5), and other five already known metabolites from an ethanolic extract of the stems of *Tinospora sinensis* (syn: *Tinospora malabarica*) (Menispermaceaeis). This plant is well known for its therapeutic value in treating debility, dyspepsia, fever, inflammation, syphilis, ulcers, bronchitis and immunomodulatory properties, as well as urinary, skin and liver diseases [53]. The five known compounds were identified as lirioresino-β-dimethyl ether, β-sitosterol, palmatine, palmatrubin and jatrorrhizine. All the compounds were assayed for antileishmanial activity against *Leishmania donovani* testing the effects of promastigotes and intracellular amastigotes, and only compound **63** exhibited the highest in vitro antileishmanial activity, whereas compounds **62**, palmatine and palmatrubin showed moderate activity. The other compounds were found to be inactive [54].

*Piper sarmentosum* and *Piper nigrum* (Piperaceae) are well known for their therapeutic effects and content of alkaloid and amides [55]. *P. nigrum* has showed CNS (central nervous system) stimulant, analgesic, antipyretic and antifeedant activities [56], while the *P. sarmentosum* leaves were used to treat malaria, coughs and colds, as well as toothache, and showed antituberculosis and antiplasmodial activities [57]. NTCT was isolated together with five known amides, namely pellitorine (*E*)-1-[30,40-(methylenedioxy)cinnamoyl]piperidine 2,4-tetradecadienoic acid isobutyl amide, piperine, sylvamide, cepharadione A and piperolactam D from *P. nigrum,* while a previously undescribed aromatic compound characterized as 1-nitrosoimino-2,4,5-trimethoxybenzene (**64**, Figure 5) was obtained from *P. sarmentosum*. The organic extracts of both plants showed cytotoxic activity against HeLa and MCF-7v cancer cell lines, with a significant contribution of compound **64** for the activity of the extract of *P. sarmentosum* [58].

NTCT and NTFT were isolated together with four previously undescribed alkaloids, namely 3-(2-(7,7-dimethyl-3,7-dihydropyrano[3,2-e]indol-1-yl)ethyl)quinazoline-2,4(1*H*,3*H*)-dione, 3-(2-(7,7-dimethyl-3,7-dihydropyrano-[3,2-e]indol-1-yl)ethyl)-1-hydroxyquinazoline-2,4(1*H*,3*H*)-dione, 3-(2-(7,7-dimethyl-3,7-dihydropyrano [3,2-e]indol-1-yl)ethyl-1-methylquinazoline-2,4(1*H*,3*H*)-dione and (*E*)-3-(6,7-dihydroxy-3,7-dimethyloct-2-enyl)-4-methoxy-1-methylquinolin-2(1*H*)-one (**65**–**68**, Figure 5), from the methanol extract of *Conchocarpus gaudichaudianus* stems (Rutaceae). This tree is used by the native people in northern Brazil [59].

**Figure 6:**
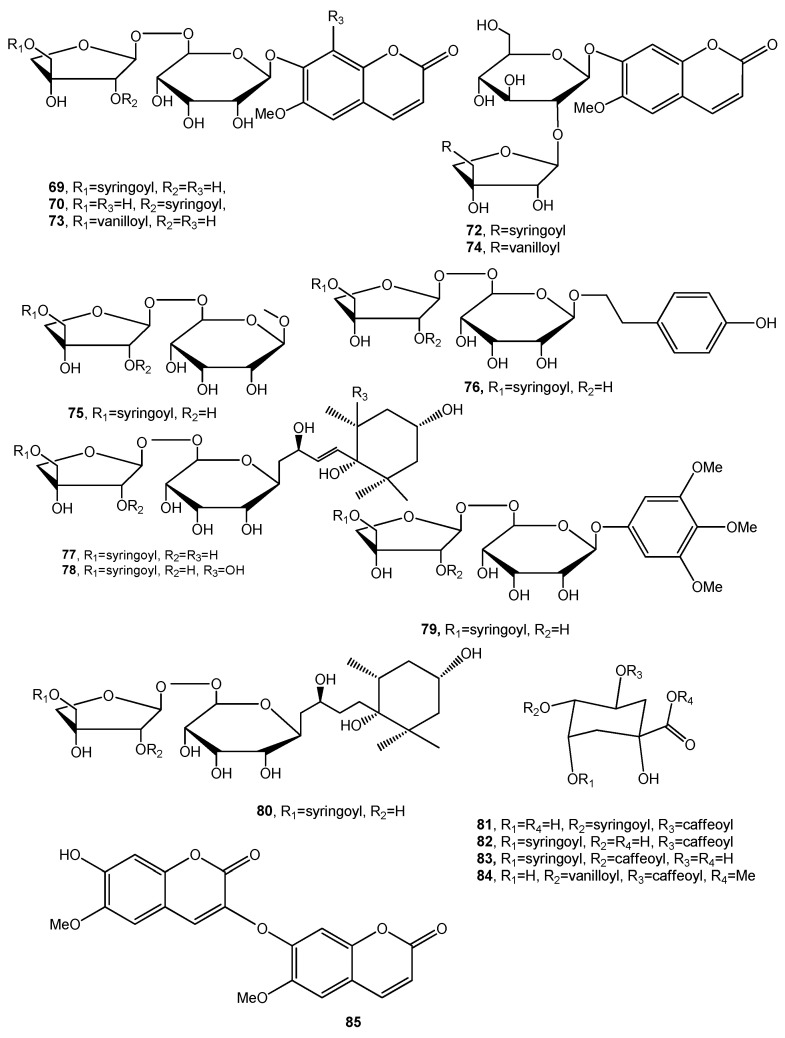
The structures of compounds **69**–**85**.

NTCT and NTFT were isolated together with 11 new diglycosides, named erycibosides A–L (**69**–**80**, Figure 6), 4 new chlorogenic acid derivatives (**81**–**84**, Figure 6), a new biscoumarin (**85**, Figure 6), and 21 other known compounds, from the roots and stems ethanol extract of *Erycibe hainanesis* (Convolvulaceae) [60]. This genus consists of about 66 species, with 11 species found in China. Compounds belonging to flavonoids, coumarins, chlorogenic acids, alkaloids and several other components were previously extracted from *Erycibe* species [61]. Some of them have been shown to exhibit anti-inflammatory, muscarinic agonistic and cytotoxic activities and have been used in Chinese folk medicine [62,63]. Erycibosides B, F and L (**70** and **74**, Figure 6) and the new biscoumarin (**85**, Figure 6) showed strong hepatoprotective activities at concentrations of 1 × 10^−5^ to 1 × 10^−4^ M [58].

NTCT and NCCT, 1,7-bis(4-hydroxyphenyl)heptane-3,5-diol and 6-hydroxy-2,4,7-trimethoxyphenanthrene were isolated from the fresh tuberous rhizomes of Chinese yam (*Dioscorea opposita* Thunb.) (Dioscoreaceae) [64]. This plant has a noteworthy interest in agriculture, food and pharmaceutical fields [65,66]. NTCT, NTCT and the hepatanediol derivative were isolated for the first time from *D. opposita*. The inhibitory activities of crude extracts as well as those of purified constituents were evaluated against yeast α-glucosidase to search for the active principles for treatment of diabetes. NTCT, the heptanediol and the phenanthrene derivative showed a significant activity with IC_50_ = 0.40, 0.38 and 0.77 μM, respectively, while NCCT was inactive suggesting that the stereochemistry of the double bond of this alkylamide is a structural feature important for the activity [64].

**Figure 7:**
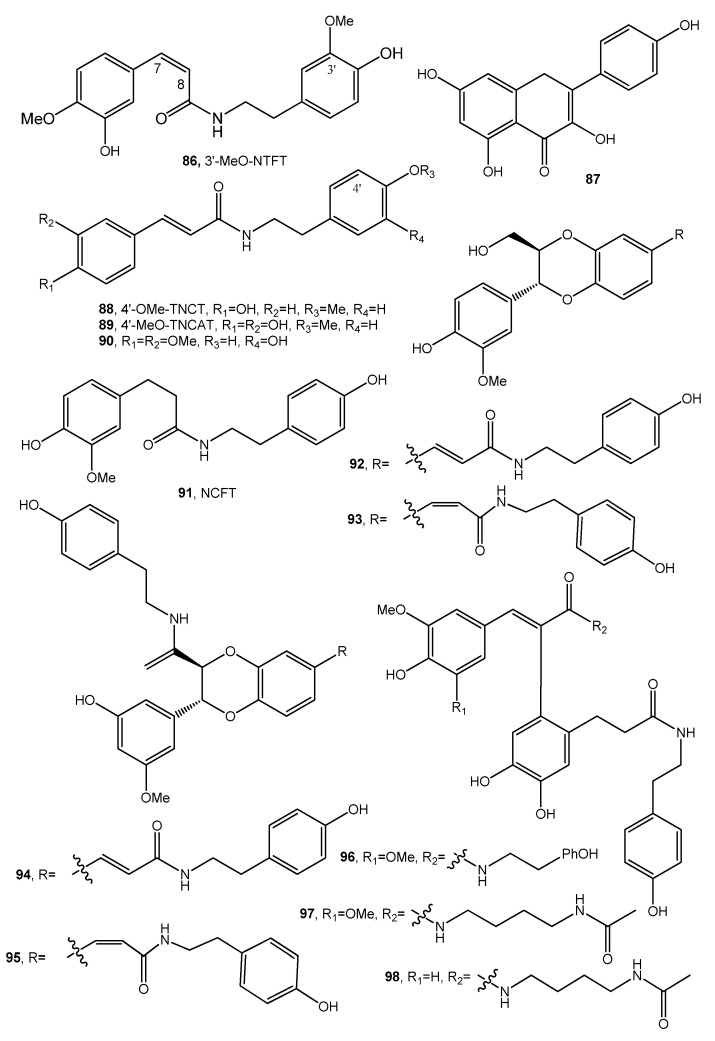
The structures of compounds **86**–**98**.

NTFT, NTCT and 3′methoxy-NTFT and kaempferol (**86** and **8**7, Figure 7) were isolated from Welsh onion (*Allium fistulosum* L.) (Amaryllidaceae) organic extracts [67]. *A. fistulosum* L. is a perennial herb that is classified as an Allium species, which is a popular flavoring vegetable in China, Japan and Korea [68]. This plant is widely cultivated in southern areas of Korea and is traditionally used for salads and cooking. In the same country, its roots and trunks were used in traditional folk medicine for the treatment of febrile disease, headache, abdominal pain, diarrhea and habitual abortion [69]. Successive studies reported that Welsh onion showed anti-aggregation [70,71] and anti-hypertensive [70,71,72,73,74] activities. The three alkylamides NTFT, NTCT and *N*-*cis*-feruloyl-3’-methoxytyramine were isolated for the first time from the Welsh onion. NTFT and its 3′-methoxy analogue exhibited significantly (*p* < 0.05) higher DPPH (2,2-diphenyl-1-picrylhydrazyl) radical scavenging activity than the compound NTCT [67].

NTCT and NTCAT were isolated together their 4′-*O*-methyl derivatives (**88** and **89**, Figure 7), β-sitostenone, ferulic, hydroferulic, 5-hydroxy-3,4-dimethoxycinnamic veratic, vanillic, isovanillic and syringic acids, as well as (+)-syringaresinol and pheophorbide D from the stems of *Capsicum annuum* (Solanaceae) [75]. Compound **88** was isolated for the first time as a naturally occurring compound [75].

NTCT, NCCT and NTFT were isolated together with ferul aldehyde, 6,7-dimethoxycoumarin and ficusal from the organic extract of *Solanum melongena* L. (Solanaceae) root [76]. The roots of this plant, called “Qie gen” in China, were used in folk Chinese medicine for the treatment of toothache, chilblains and beriberi. Other studies showed that the extracts of *S. melongena* had anti-inflammatory, analgesic and antiatherosclerosis activities [77,78]. Only the three alkylamides NTCT, NCCT and NTFT inhibited α-glucosidase with IC_50_ values of 500.6, 5.3 and 46.3 μM, respectively, and they were not competitive inhibitors. Thus, the plant could be proposed for pharmacological application [76].

NTCT, NTFT and NTCAT were isolated as the main component from the organic extract of *Polygonum hyrcanicum* (Polygonaceae) aerial parts, which showed high activity against *Trypanosoma brucei rhodesiense* (IC_50_ = 3.7 μg/mL). This protozoan parasite induces sleeping sickness, also known as human African trypanosomiasis (HAT). HAT infects more than 50,000 people each year and about 60 million people are at risk of trypanosomiasis [79]. The three alkylamides, NTCT, NTFT and NTCAT, showed activity with C50s ranging from 2.2 to 13.3 μM [80]. *P. hyrcanicum* is an endemic plant growing in northern areas of Iran and is known as Gheq-buqun in the Turkmen Sahra region, where its decoction has been used for the treatment of liver problems, anemia, hemorrhoids and kidney stones [81]. From the same organic extract, some other known and lesser active compounds were also isolated as cannabisin B, tyrosol, *p*-coumaric and ferulic acids, and NCFT and *N*-*trans*-3,4-dimethoxycinnamoyldopamine (**90**, Figure 7). This data again showed that *E* stereoisomer is more active than the *Z* one (NCFT). However, it is important to remember that cinnamoylphenethyl amides rapidly isomerize when exposed to UV light and therefore NCFT could be an artifact formed during the isolation procedure [82].

**Figure 8:**
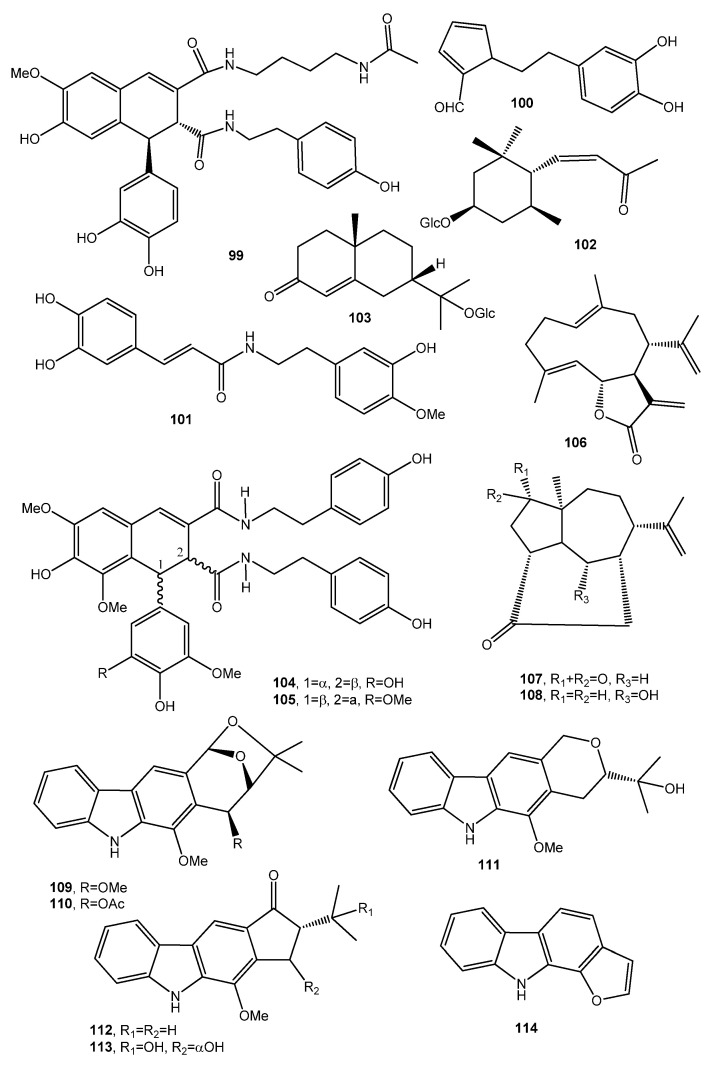
The structures of compounds **99**–**114**.

NTCT, NTFT, NTCAT and *N*-*cis*-feruloyloctopamine (NCFO (**91**, Figure 7)) were isolated together with 7 new neolignanamides (**92**–**98**, Figure 7), a new lignanamide (**99**, Figure 8) and 17 known phenolic compounds from the organic extract of *Lycium chinense* [83]. This plant belongs to genus *Lycium* (Solanaceae family) mainly distributed in South America, South Africa and temperate Europe and Asia. It was used in traditional Chinese medicine as an antipyretic and for the treatment of pneumonia, night-sweats, cough, hematemesis, inflammation and diabetes mellitus [84]. The known compounds were identified as thoreliamide B, gentisic, vanillic, *p*-coumaric caffeic, ferulic, sinapic and dihydrocaffeic acids, as well as isoscopoletin, fraxidin, aquillochin, scopolin, kaempferide, apigenin and luteolin. The cinnamic acid amides, neolignanamides and lignanamides showed moderate radical scavenging activity towards the DPPH and superoxide radicals [83].

NTCT and NTFT were isolated from the organic extract of *P. oleracea* (Portulacaceae) together with a pyrrole alkaloid named portulacaldehyde (**100**, Figure 8), *N*-(*E*)-feruloyl-4-*O*-methyldopamine (**101**, Figure 8) and the well-known (*E*)-*p*-coumaric and (*E*)-ferulic acids, 4-hydroxybenzaldehyde, 2,4-dihydroxybenzaldehyde, 2-hydroxy-4-methoxybenzoic and syringic acids [85]. *P. oleracea*, commonly named purslane, is an annual, semi-succulent, trailing herbaceous plant used in folk medicine for its analgesic and wound-healing, anti-inflammatory properties [86,87]. *N*-(*E*)-feruloyl-4-*O*-methyldopamine (**101**), NTFT, 4-hydroxybenzaldehyde and 2,4-dihydroxybenzaldehyde were involved in the regulation in the inflammatory activity of the plant extract [83].

NTCT, NCCT, NTFT and NCFT were isolated together with 13 megastigmanes, including a new megastigmane, nelumnucifoside A (**102**, Figure 8), and a new eudesmane sesquiterpene, nelumnucifoside B (**103**, Figure 8), as well as 8 alkaloids and 11 flavonoids from *Nelumbo nucifera* Gaertn. (Nymphaeaceae) [88]. This is a perennial aquatic herb commonly called lotus. This plant is widely diffused in Eastern Asia and used for food and medicine for a long time. The fruits, seeds, roots and leaves of *N. nucifera* are edible and have been not only used as food for a long time, but also used as antifebrile, sedative, antibacterial, antidiarrheal and hemostatic agents in folk medicine [89]. The other known compounds were identified as (*E*)-3-hydroxymegastigm-7-en-9-one, (−)-boscialin, (+)-dehydrovomifoliol, vomifoliol, 3-oxo-retro-α-ionol I, byzantionoside A, 5,6-epoxy-3-hydroxy-7-megastigmen-9-one, annuionone D, icariside B_2_, grasshopper ketone, 3*S*,5*R*-dihydroxy-6*S*,7-megastigmadien-9-one, (+)-epiloliolide, (6*R*,6a*R*)-roe-merine-*N*_β_-oxide, liriodenine, pronuciferin, oleracein E, quercetin, kaempherol, luteolin, quercetin 3-*O*-glucopyranoside, kaempherol 3-*O*-glucopyranoside, chrysoeriol 7-*O*-glucopyranoside, taxifolin, epitaxifolin, 5,7,3′,5′-tetrahydroxyflavanone, (−)-catechin and elephantorrhizol. NTCT and NCFT inhibited pancreatic lipase, while (6*R*,6a*R*)-roemerine-*N*_β_-oxide and liriodenine showed a strong inhibition on adipocyte differentiation. Therefore, the extract of *N. nucifera* leaves has potential as an anti-obesity agent [88].

NTCAT, NTFT, NTCT and *N*-*trans*-feruloyldopamine were isolated together with the well-known 13-hydroxysolavetivone, betulinic acid, 3′-*O*-methoxydopamine, alangilignoside C, isolariciresinol, polistachiol, (+)-(8*R*,7′*S*,8′*S*)-3α-*O*-(β-d-glucopiranosyl)-lioniresinol, (−)-(8*S*,7′*R*,8′*R*)-3α-*O*-(β-d-glucopiranosyl)-lioniresinol and solamargine from the organic extract of *Solanum buddleifolium* Sendtn (Solanaceae) stems [90]. *S. buddleifolium* is widely distributed in the northeast of Brazil, where it is used in folk medicine [91].

NTFT was isolated together with two *bis*-alkaloids, flavifloramides A and B (**104** and **105**, Figure 8), and paprazine from the aerial part of *Piper flaviflorum* [92]. This plant belongs to the *Piper* genus, which is well known as a rich source of a variety of alkaloids, having interesting pharmacological activities, such as anti-inflammatory, antino-ciceptive, anticancer and antidepressant properties [92,93,94].

*N*-*tran*s-Cinnamoyltyramine (**1**, Scheme 3) and NTCT were isolated together with two sesquiterpenes, named aristoyunnolins I and J (**106** and **107**, Figure 8), and six other known compounds from the roots of *Aristolochia yunnanensis* (syn. *Aristolochia griffithii*) (Aristolochiaceae) [95]. This plant is endemic to Yunnan Province of China, known as “Nan Mu Xiang”, and is used in Chinese medicine for the treatment of trichomoniasis, gastrointestinal diseases and rheumatic pain [94]. All the compounds were evaluated against P-388 and A-549 cell lines, and among them costunolide (**108**, Figure 8) exhibited moderate activity [95].

NTCT, NTFT, NTCAT, dihydro-NTCAT and three neolignanamides and two lignanamides were isolated from the root bark of *Lycium chinense* Miller, Lycii Radicis Cortex (Solanacee). This plant was used in traditional Chinese medicine to treat different inflammation symptoms and diabetes mellitus [96]. The results of biological assays showed that akylamides, as main components of *L. chinese,* were responsible for NF-κB inhibition. The SAR study also suggests that the NF-κB inhibitory activity of NTCAT could be due to its Michael acceptor-type structure (α,β-unsaturated carbonyl group) [97].

**Figure 9:**
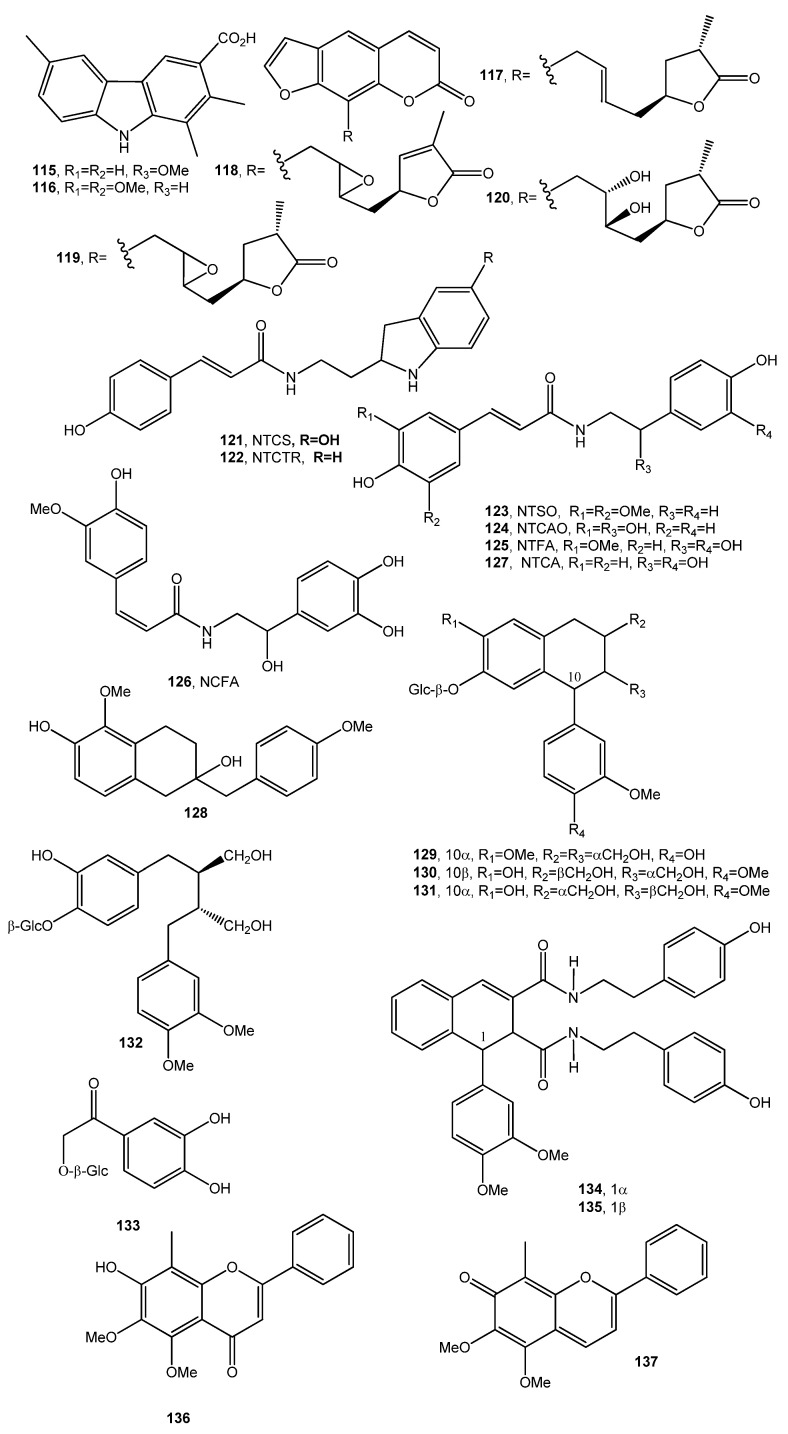
The structures of compounds **115**–**137**.

NCCT, NTCT, 8 carbazole alkaloids, claulamines C, D and E (**109**–**111**, Figure 8) and clausenalines B−F (**112**–**114**, Figure 8, **115**–**116**, Figure 9), as well as 4 coumarins, clausemarins A−D (**117**–**120**, Figure 9), and 41 already known compounds were isolated from the roots of *Clausena lansium* (Rutaceae) [98]. This plant, also known as “wampee”, is a native species of southern mainland China and it was used in folk medicine in China, Taiwan and the Philippines. Its leaves and roots are used for coughs, asthma, dermatological diseases, viral hepatitis and gastrointestinal ailments [99], while the seeds are used for acute and chronic gastrointestinal inflammation and ulcers [100]. Several known compounds were also identified as wampetin, 8-geranyloxypsolaren, imperatorin, osthenol, isoimperatorin, *O*-demethylmurrayanine *O*-demethylmurrayanine, clausine D, methyl carbazole-3-carboxylate, murrayanine, *O*-methyllansinexanthotoxol, heraclenol, anisolactone, claulansine A, *O*-methylmukonidine, 3-formyl-9*H*-carbazole, claulansine F, claulansine C, 9*H*-carbazole-3-carboxylic acid, 1-methoxycarbazole-3-carboxylic acid, 4-methoxy-1-methyl-2(1*H*)-quinolinone, vanillic acid, 2,6-dimethoxy-*p*-benzoquinone, 4-hydroxybenzoic acid, *N*-phenethylcinnaamide, (*E*)-coniferaldehyde, claulansine J, 3-formyl-6-methoxycarbazole, tertmethoxyheraclenol, isogospherol, indicolactonediol, lucidafuranocoumarin B, mafaicheenamine C, syringaresinol, mafaicheenamine A, mukonine, dihydroalatamide, α-santalol, β-sitosterol, platydesmine and γ-fagarine. Clausemarin A (**117**), wampetin, 8-geranyloxypsolaren, imperatorin, osthenol, isoimperatorin and *O*-demethylmurrayanine showed strong inhibition of superoxide anion generation with IC_50_ values ranging from 1.9 to 8.4 μM, while compounds *O*-demethylmurrayanine, clausine D and murrayanine inhibited elastase release with IC_50_ values in the range from 2.0 to 6.9 μM [98].

NTCT and NTFT were isolated together with the well-known 4-hydroxybenzaldehyde, *N*-*p*-coumarylserotonine (NTCS, **121**, Figure 9) and *N*-*p*-coumaryl-tryptamine (NTCTR, **122**, Figure 9) from the stem of *Zea mays*, which is cultivated worldwide as grain and feed, and its seeds, oil, stigma, spike, leaf and root have been used in Chinese traditional medicines. *Z. mays* chloroformic extract showed antiacetylcholinesterase activity [101].

NTCT and NCCT were isolated together with the already known methyl-10,10-dimethoxydecanoate, methyl-10-hydroxy-8*E*,12*Z*-octadecadienoate, methylcoriolate, *trans*-phytol, phytene-1,2-diol, phyton, (3*S*,5*R*,6*S*,7*E*,9*R*)-7-megastigmene-3,6,9-triol, (3*S*,5*R*,6*S*,9*R*)-3,6,9-trihydroxymegastigman-7-ene, shikimic acid, *p*-coumaramide, tryptophan, thymidine, adenosine and deoxyadenosine from the aqueous methanol extract of *Hosta longipes* (Liliaceae) [102]. This is an edible plant widely distributed in Korea, China and Japan and has been used in traditional Korean medicine for treating cough, laryngopharyngitis, burns, swelling, snake bites and inflammation.

Further studies on the chemical metabolites produced by *S. melongena*, in addition to the three alkylamides NTCT, NCCT and NTFT [76] reported above, showed that it also produced other interesting amides. In particular, *N*-*trans*-sinapoyloctopamine (NTSO), *N*-*trans*-caffeoyloctopamine (NTCAO), *N*-*trans*-feruloylnoradrenline (NTFA) and *N*-*cis*-feruloylnoradrenline (NCFA) (**123**–**124**, **126**, Figure 9) were isolated for the first time as naturally occurring compounds together with the already known 3-(4-hydroxyphenyl)-*N*-[2-(4-hydroxyphenyl)-2-methoxyethyl] acrylamide, 3-(4-hydroxy-3-methoxyphenyl)-*N*-[2-(4-hydroxyphenyl)-2-methoxyethyl] acrylamide and *N*-*trans*-*p*-coumaroylnoradrenline (NTCA, **127**, Figure 9) [103].

NTFT, NTCT, NCFT and NTFO were isolated together with (3*R*)-3,7-dihydroxy-8-methoxy-3-(4′-methoxybenzyl)-4-chromanone (**128**, Figure 9), four flavonoids, three steroids, pinoresinol and lanost-9-en-3β-ol from the leaves of *Dracaena cochinchinensis* (Lour.) S. C. Chen (Asparagaceae). The four flavonoids and the three steroids were identified as (2*S*)-4′, 7-dihydroxy-3′-methoxy-8-methylflavan (2*S*)-3′,7-dihydroxy-4′-methoxy-8-methylflavan, 7-hydroxy-3-(4′-methoxybenzyl)-4-chromanone and 2′,4′,4-trihydroxychalcone and (22*E*)-3β-acetoxystigmasta-5,22-diene, β-sitosterol and β-daucosterol, respectivelt [104].

NTCT was isolated together with 5 phenolic glycosides, named sargentodosides A-E (**129**–**133**, Figure 9), 2 dihydronaphthalene lignans, named sargentodognans F and G (**134** and **135**, Figure 9) and 31 known phenolic compounds from the ethanolic extract of *Sargentodoxa cuneata* (Oliv.) Rehd. Et Wils (Lardizabalaceae) [105]. This plant is diffused in south, east, central and southwest China, and its stems are used in Chinese folk medicine for the treatment of rheumatic arthritis, abdominal pain, acute appendicitis, trauma, dysmenorrhea, amenorrhea and painful menstruation. The known compounds were identified as (+)-isolariciresinol-9′-*O*-β-d-glucopyranoside, slvadoraside, glehlinoside C7-hydroxy-1-(4-hydroxy-3-methoxyphenyl)-*N*_2_,*N*_3_-bis(4-hydroxyphenethyl)-6-methoxy-1,2-dihydro-naphthalene-2,3-dicarboxamide, sargentol, cuneataside C, osmanthuside H, crosatoside B, echipuroside A, 6-(β-d-glucopyranosyloxy)-2*R*,4-dihydroxy-2-[(4-hydroxyphenyl)methyl]-3(2*H*)-benzofuranone, 6-(β-d-glucopyranosyloxy)-2*S*,4-dihydroxy-2-[(4-hydroxyphenyl)methyl]-3(2*H*)-benzofuranone, 1-*O*-α-rhamnopyranosyl-(1″→6′)-*O*-β-d-glucopyranosyl-2-methoxy-4-acetylphenol, 1-*O*-α-L-rhamnosyl(1″-6′)-β-d-glucopyranosyloxy-3,4,5-trimethoxybenzene, 4-*O*-β-d-glucopyranosyl-3-hydroxylbenzoic acid, protocatecheuic acid 3-*O*-β-d-glucoside, caffeic, protocatechuic, vanillic and 3-*O*-caffeoylquinic acids, catechin, (−)-epicatechin, dulcisflavan, cinchonains Ia, hydroxytyrosol, acid, calceolarioside B, 2-(4-hydroxyphenyl)ethyl-[6-*O*-(*E*)-caffeoryl]-*O*-β-d-glucopyranoside, salidroside, 2-(3,4-dihydroxyphenyl)ethyl-*O*-β-d-glucopyranoside, icariside D2, methyl 3-*O*-caffeoylquinate and procyanidin B-2 [105]. Catechin, (−)-epicatechin, dulcisflavan, cinchonains Ia, caffeic acid, 2-(4-hydroxyphenyl)ethyl-[6-*O*-(*E*)-caffeoryl]-*O*-β-d-glucopyranoside, 2-(3,4-dihydroxyphenyl)ethyl-*O*-β-d-glucopyranoside and methyl 3-*O*-caffeoylquinate showed antibacterial activities against *Staphylococcus aureus* ATCC 29213 with MIC values in the range of 2–516 μg/mL. Hydroxytyrosol showed the highest activity against the same bacterium with an MIC value of 2 μg/mL, while no compound exhibited antimicrobial activities against *C. albicans* ATCC 10231. Sargentol, cinchonains Ia and 2-(3,4-dihydroxyphenyl)ethyl-*O*-β-d-glucopyranoside significantly inhibited the proliferation in the two cancer cell lines as Hela and Siha, showing stronger activity than cisplatin in the cytotoxic assay [105].

**Figure 10:**
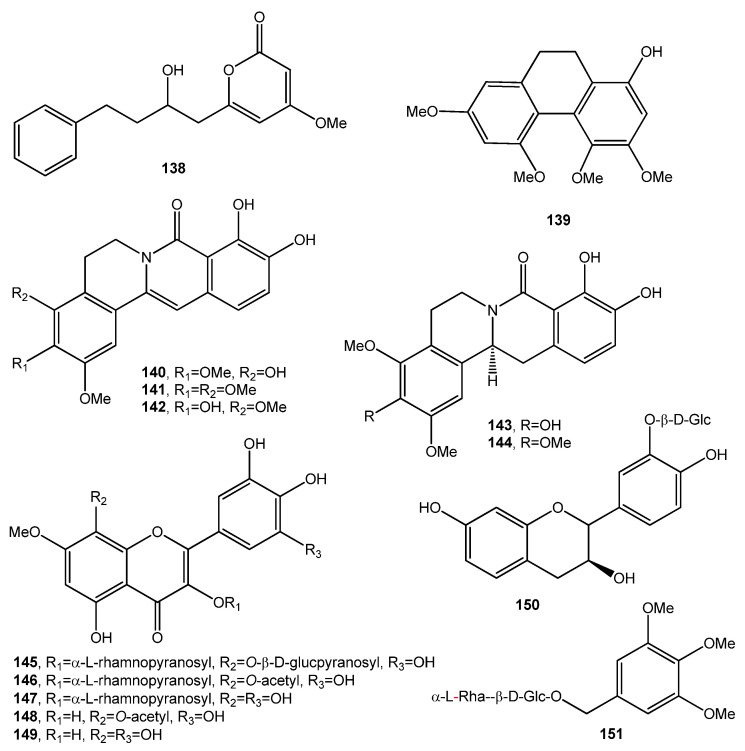
The structures of compounds **138**–**151**.

NTCT, NTFT, NTCO and NTFO, were isolated together with two C-methylated flavonoids, namely 5,6-dimethoxy-7-hydroxy-8-methyl-flavone and 5,6-dimethoxy-8-methyl-2-phenyl-7*H*-1-benzopyran-7-one (**136** and **137**, Figure 9), and an α-pyrone, namely 4-methoxy-6-(2-hydroxy-4-phenylbutyl)-2*H*-pyran-2-one (**138**, Figure 10). They were also isolated with 13 known compounds, including five amides, from *Talinum triangulare* (Portulacaceae) [106]. This plant, probably native to tropical America, was introduced to Nigeria and other tropical regions in Africa as a leaf vegetable. Now it is one of the most important vegetables in Nigeria known as the “waterleaf” [107]. However, its leaves were also used for the treatment of peptic ulcer, cuts, wounds and scabies, and the roots’ decoction for hypertension [108,109]. The other known compounds were identified as cannabisin F, grossamide, aurantiamide, aurantiamide acetate, aurantiamide benzoate, indole-3-carboxylic acid, *p*-hydroxy benzoic acid, 3β-hydroxystigmast-5,22-dien-7-one and 3β-hydroxystigmast-5-en-7-one. Any compound showed cytotoxic activity against L5178Y mouse lymphoma cell line [106].

NTCT was isolated together with 9,10-dihydrophenanthrene-1,5-dihydroxy-3,4,7- trimethoxy-9,10-dihydrophenanthrene (**139**, Figure 10) and 24 known compounds from the whole plants of *Dendrobium moniliforme* (Orchidaceae) [110]. This plant is widely distributed in China, India, Korea and Japan, and its constituents showed different biological activities including antitumor, anti-inflammatory, antiplatelet and anti-aggregation activities [111,112]. The other known compounds were identified as ashircinol, (2R*,3S*)-3-hydroxymethyl-9-methoxy-2-(4′-hydroxy-3′,5′-dimethoxyphenyl)-2,3,6,7-tetrahydrophenanthro[4,3-b]furan-5,11-diol, diospyrosin, aloifol I, moscatilin, 3,4′-dihydroxy-3′,4,5-trimethoxybibenzyl, gigantol, 3,3′-dihydroxy-4,5-dimethoxybibenzyl, longicornuol A, paprazine, *N*-*trans*-feruloyl 3′-*O*-methyldopamine, moupinamide, dihydroconiferyldihydro-*p*-coumarate, dihydrosinapyl dihydro-*p*-coumarate, 3-isopropyl-5-acetoxycyclohexene-2-one-1, *p*-hydroxybenzaldehyde, vanillin, *p*-hydroxyphenylpropionic, vanillic and protocatechuic acids , (+)-syringaresinol, β-sitosterol and daucosterol [110].

NTCT, NTFT and NCFT were isolated together with 12 known compounds from sweet potato (*Ipomoea batatas*) leaf. The other known compounds were identified as 3,4,5-tricaffeoylquinic (3,4,5-triCQA), 3,4-dicaffeoylquinic (3,4-diCQA), 3,5-dicaffeoylquinic (3,5-diCQA), 4,5-dicaffeoylquinic (4,5-diCQA), 4,5-feruloylcourmaoylquinic and caffeic acids, caffeic acid ethyl ester, 7-hydroxy-5-methoxycoumarin, quercetin-3-*O*-α-d-glucopyranoside, 7,3′-dimethylquercetin, rhamnetin and indole-3-carboxaldehyde. NTCT, NTFT, NCFT and 3,4,5-triCQA showed the strongest α-glucosidase inhibition, while 3,4,5-triCQA and diCQAs were the dominant antioxidants. The results of a SAR study demonstrated that higher caffeoylation of quinic acid and lower methoxylation of flavonols induced stronger antioxidant activity, while methylation and *cis*-configuration of phenethyl cinnamides weaken the α-glucosidase inhibition [113].

NTFT, NTCAT and NTCT were isolated from the leaves *Miliusa cuneata* (Annonaceae) organic extract together with five oxoprotoberberine alkaloids, named miliusacunines A–E (**140**–**144**, Figure 10). The twig extract of the same plant allowed researchers to identify five known metabolites as 5-hydroxy-3,7-dimethoxy-3′,4′-methylenedioxyflavone, pachypodol, 4′-hydroxy-3,5,7,3′-tetramethoxyflavone, (+)-miliusol and (+)-syringaresinol [114]. This plant as well as others belonging to the same genus are distributed from the Indian subcontinent to Indochina, the Malaysia Peninsula and the southeast Asian islands, to New Guinea and northern Australia. Some species are used in traditional medicine as a tonic and aphrodisiac and for gastropathy. All the compounds were assayed for cytotoxic activity against KB and Vero cancer cell lines and for antimalarial activity against the *Plasmodium falciparum*. Miliusacunine A (**138**) showed in vitro antimalarial activity against the TM4 strain, with an IC_50_ value of 19.3 ±3.4 μM, while miliusacunine B (**139**) exhibited strong activity against the K1 strain, with an IC_50_ value of 10.8 ± 4.1 μM. No compound showed cytotoxic activity [114].

NTFT and NTCT were isolated together with 5 7-methoxyflavonols with pyrogallol B-ring moieties (**145**−**149**, Figure 10), a fisetinidol glucoside (**150**, Figure 10), a benzyl glycoside (**151**, Figure 10), and 23 known compounds [115] from *Atraphaxis frutescens* (L.) K. Koch (Polygonaceae). This is a shrub found in the Mongolian Gobi [116] and its dried aerial parts are used in traditional Mongolian medicine for detoxification and to treat lymph disorders, bacterial fevers, throat infections and eye diseases, including cataracts [117]. The known compounds were identified as europetin 3-*O*-α-l-rhamnopyranoside, myricitrin, fisetinidol, gallocatechin, catechin, afzelechin, aromadendrin, epigallocatechin, epicatechin, nikoenoside, emodin 8-*O*-β-d-glucopyranoside, emodin 8-*O*-(6′-*O*-malonyl)glucoside, torachrysone 8-*O*-β-d-(6′-*O*-malonyl) glucopyranoside, syringaresinol, dehydroconiferyl alcohol, 3,4,5-trimethoxyphenyl 1-*O*-β-d-glucopyranoside and methyl syringate [115]. Compounds containing either a pyrogallol or a catechol B-ring moiety showed potent radical scavenging activity, while insect phenoloxidase and mushroom tyrosinase were, respectively, inhibited by phenylpropanoid amides and by the characteristic 7-methoxyflavonol-3-*O*-rhamnopyranosides [115].

**Figure 11:**
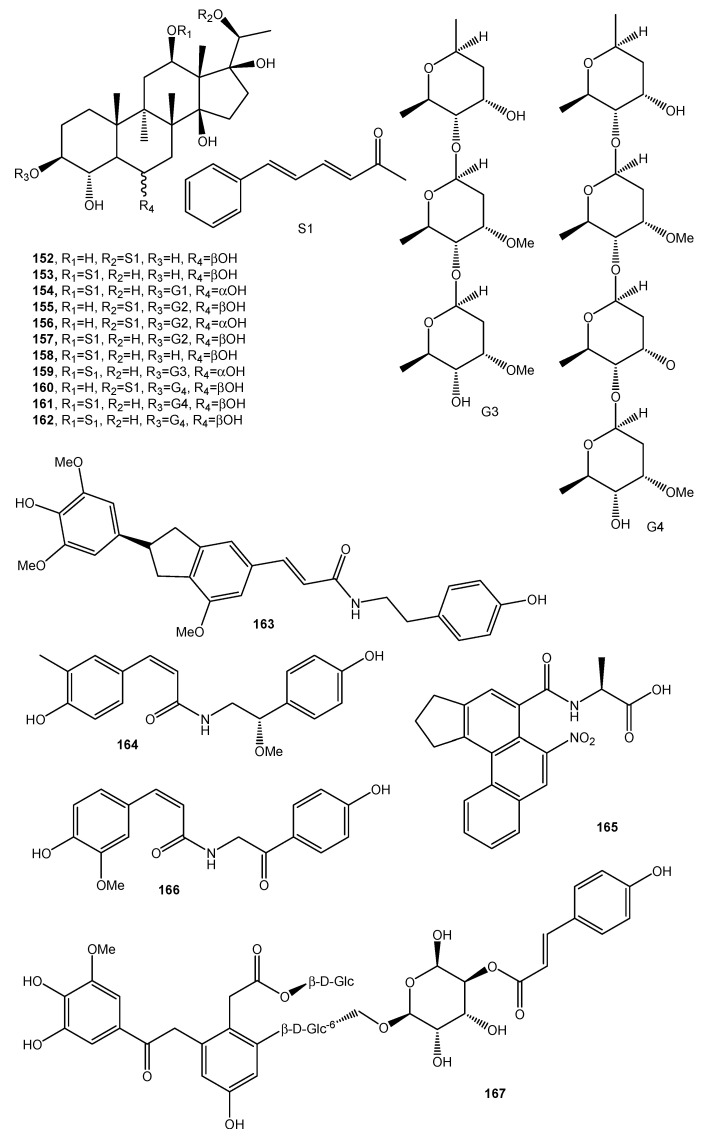
The structures of compounds **152**–**167**.

NCFT, NTCAT and NTCT were isolated together with 11 new octahydroxylated C_21_ steroids, named with lyciumsterols A–K (**152**–**162**, Figure 11), and 13 already known compounds from the root bark of *Lycium chinense,* a plant used in Chines folk medicine as described above. Lyciumsterols B, C and G (**153**, **154**, and **157**) showed protective effects on pancreatic islet cells but were dose dependent, while lyciumsterols G–I and K, (**158**–**160** and **162**) exhibited autophagy activation [118].

NCCT and NTCT were isolated together with 13 already known compounds from *Coixlachryma*-*jobi* var. *mayuen* (Gramineae). The already known compounds were identified as (7*R*,8*S*)-3′-demethyl-dehydrodiconiferyl alcohol-3′-*O*-β-glucopyranoside, (*7R*,8*S*)-3′-demethyl-9′-butoxy-dehydrodiconiferyl-3′-*O*-β-glucopyranoside, adenosine 2-*O*-caffeoyl isocitricacid, pseudolaroside, 2-hydroxy-7-methoxy-(2*H*)-1,4-benzoxazin-3(4*H*)-one, 2-*O*-β-glucopyranosyl-7-methoxy-2*H*-1,4-benzoxazin-3(4*H*)-one, 2-*O*-β-glucopyranosyl-4-hydroxy-7-methoxy-2*H*-1,4-benzoxazin-3(4*H*)-one, 2-*O*-β-d-glucopyranosyl-7-hydroxy-2*H*-1,4-benzoxazin-3(4*H*)-one, *p*-coumaric acid and caffeic acid ethyl ester, *p*-coumaric acid and coixol [119]. *C.*-*joby* var. *mayuen,* which is an annual, tropical plant native of Asia, namely from India to peninsular Malaysia, and it is now diffused in southeast Asia and the USA, is used in folk Chinese medicine to treat inflammation, dysfunctions of the endocrine system, chapped skin, warts, arthritis and neuralgia [120].

NTCT, NTFT, NTCAT and NCFT were isolated together with two new phenolic amides, characterized as (7*R*,8*S*)-7-(4-hydroxy-3,5-dimethoxyphenyl)-8-hydroxy methyl-10-[*N*-7″-(4″-hydrxyphenyl)ethyl]carbamoylethenyl-3′-methoxybenzodihydrofuran and *cis*-*N*-*p*-hydroxycinnamoyl-7′-methoxyethyltyramine (**163** and **164**, Figure 11), together with eight known compounds from *Nicandra physaloides* (Solanaceae) [121]. This is an annual herb native to Peru, but it is diffused in Yunnan, Guangxi, Guizhou and some other Chinese provinces, where it is used in traditional folk medicine as sedative, expectorant, antipyretic and as an antidote. Its leaf extracts induced decrease blood sugar but also showed antitumor and insect antifeedant properties [122,123,124]. The other known compounds were identified as *trans*-*N*-feruloyloctopamine (NTFO), *trans*-*N*-feruloyl-7′-methoxyltyramine, cannabisin D, grossamide K, *trans*-*N*-hydroxycinnamoyl-7′-methoxyltyramine, erythro-canabisine H and cannabisin E. NCFT, NTFO and NTCAT showed significant protective activities on 1-methyl-4-phenylpyridiniumion (MPP+)-induced damage in human dopaminergic neuroblastoma cells (SH-SY5Y). The cell protection mechanism of NCFT was due to its ability to inhibit apoptosis and inducing cytoprotective autophagy in Parkinson’s disease (PD) [121].

NTCT, the aristolochic acid II alanine amide (**165**, Figure 11) and other known compounds were isolated from *Aristolochia maurorum* (Aristolochiaceae) [125]. This latter is a perennial herb that widely grows in Jordan [126]. The other known compounds were identified as palmitic acid, β-sitosterol, *E*-ethyl-*p*-coumarate, *Z*-ethyl-*p*-coumarate, aristolochic acid IV methyl ester, aristolactam I, loliolide, (+)-dehydrovomifoliol, glycerol-1-palmitate, aristolochic acid I, *E*-*p*-coumaric acid, β-sitosterylglucoside, aristolochic acid IV, aristolochic acid III, esculetin, uracil, shepherdine and adenosine [125].

NTFT and NTCT were isolated together with phenolic amide, named *cis*-terrestriamide (**166**, Figure 11), and seven known compounds from the fruits’ organic extract of *Tribulus terrestris* (Zygophyllaceae) [127]. This is an annual creeping plant is widely diffused in tropical regions, including Korea, China and Japan, and its fruits have been used in folk medicine to treat dizziness, headache, high blood pressure, menstrual irregularity, pruritus, eye problems, edema, abdominal distention, sexual dysfunction and cardiovascular diseases [128]. The known compounds are essentially the alkylamides *N*-*trans*-cinnamoyltyramine (**1**), *N*-*trans*-feruloyloctopamine and *N*-(2-(4-hydroxyphenyl)-2-methoxyethyl)cinnamamide, terrestriamide and ferulamide [127].

NTCT, NTFT, NCCT, NCFT and a flavonoid glucoside, named ruthenicunoid A (**167** Figure 11), were isolated together with five known compounds from the fruits of *Lycium ruthenicun* Murr. (Solanacea) [129]. This plant is diffused in the northwest regions of China, and its edible fruits are used for the treatment of hypertension, ureteral stones, tinea, furuncle and gingival bleeding [130,131,132]. The other known compounds were identified as *N*_1_,*N*_10_-bis(dihydrocaffeoyl)spermidine, *N*-*trans*-feruloyl-3′-*O*-methyldopamine *N*-*trans*-feruloyloctopamine (NTFO) and *N*-*cis*-feruloyloctopamine (NCFO) [129]. Ruthenicunoid A (**167**) and *N*_1_,*N*_10_-bis(dihydrocaffeoyl)spermidine showed the concentration-dependent inhibition of SIRT1 (full-length human protein/cytokine/chemokine/growth factor) [129].

**Figure 12:**
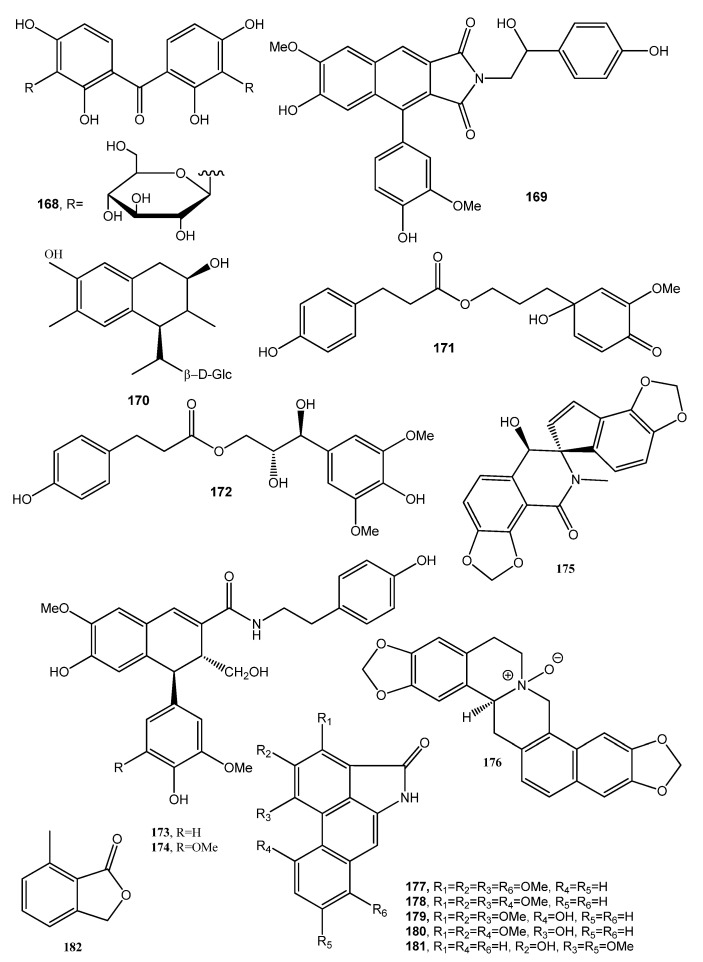
The structures of compounds **168**–**182**.

NTFT, NTCT and the benzophenone C-glucoside, named pseuduvarioside (**168**, Figure 12), were isolated together with four known compounds from the leaves and stems of *Pseuduvaria fragrans* Y. C. F. Su, Chaowasku and R.M.K. Saunders (Annonaceae) [133]. This species was collected in peninsular Thailand [134]. The other known compounds were identified as (−)-guaiol, (+)-isocorydine, cyathocaline and isoursoline. NTFT and NTCT were noncompetitive inhibitors of α-glucosidase [133].

NTFT and NTCT were isolated together with an isoindole alkaloid, named oleraisoindole (**169**, Figure 12), together with four known compounds, from *Portulaca oleracea* L. (Portulacaceae) [135]. The known compounds were identified as 7′-ethoxy-*trans*-feruloyltyramine, *N*-*trans*-feruloyl-3-methoxytyramine, aurantiamide and ferulic acid methyl ester. Oleraisoindole (**169**) inhibited NO production in RAW 264.7 cells induced by LPS [135].

NTCT and cadinane sesquiterpenoid glucoside, characterized as 2β,7,3-trihydroxycalamenene 3-*O*-β-d-glucoside (**170**, Figure 12) were isolated together with five known compounds from the stem bark of *Abelmoschus sagittifolius* (Malvaceae) [136]. This plant is considered an edible food in Hainan Island of China and southeast Asian countries and widely used in folk medicine for the treatment of phthisis, cough, constipation, neurasthenia, carbuncle sore swollen poison, dizziness and lumbocrural and stomach pains. The already known compounds were identified as *N*-(*p*-*trans*-coumaroyl)-*N*-methyltyramine, cleomiscosin A, 9,12,13-trihydroxy-10,15-heptadecadienoic acid, cytochalasin B and marmesinin. All the isolated metabolites showed moderate cytotoxicity against Hela and HepG-2 human cancer cell lines [136].

NTCT and two new phenylpropanoid esters, named bobulretulates A (**171** and **172**, Figure 12) were isolated together with 10 known compounds from the whole plants of *Bulbophyllum retusiusculum* (Orchidaceae) [137]. This plant is widely diffused in China, Nepal, Sikkim, Bhutan, India, Burma, Laos and Vietnam. The other already known compounds were identified as paprazine, dihydro-feruloyltyramine, guaiacylglycerol, erythro-guaiacylglycerol, 4-(2-hydroxyethyl)-2-methoxyphenyl-β-d-glucopyranoside, thymidine, uridine, roseoside, 6,9-dihydroxy-4,7-megastigmadien-3-one and β-sitosterol [137].

NTCT, NTFT and NTCAT were isolated together with 17 already known compounds, including three sterols, three phenols, four anthraquinones, one chromone, two stilbenes, three flavonoids and one organic acid from *Fallopia convolvulus* (L.) A. Löve (Fallopia) [138]. This is an annual herbaceous plant distributed in different Chinese districts, and its roots were used to treat inflammation, insomnia, infection and arthritis. The known compounds were identified as stigmast-4-en-3-one, stigmast-4-en-3,6-dione, stigmast-4-en-3β,6α-diol, ethyl-*p*-hydroxybenzoate, emodin-1,6-dimethylether, 7-hydroxy-2,5-dimethylchromone, physcion, citreorosein, *trans*-resveratrol, piceatannol, *p*-hydroxybenzaldehyde, protocatechuic acid, rhein, tricin, luteolin, myricetin and succinic acid [138].

NTCT, two lignanamides, named majusamides A and B (**173** and **174**, Figure 12), and two alkaloids, named chelidoniumine and tetrahydrocoptisine-*N*-oxide (**175** and **176**, Figure 12), were isolated together with five known hydroxycinnamic acid amides (HCCA) from *Chelidonium majus* (Papaveraceae) organic extract [139]. This plant is widely diffused in the south and northeast of China, including Inner Mongolia, Jilin, Heilongjiang, Liaoning, Henan and other places. The main active components of *C. majus* are alkaloids that exhibited analgesia, anti-inflammatory, anti-microbial, antineoplastic, insecticidal and antioxidant activity [140,141]. The already known compounds were identified as *N*-*trans*-feruloyldopamine, *N*-*trans*-feruloyl-3-methoxytyramin, (*E*)-3-(4-hydroxy-3-methoxybenzylidene)-4-(4-hydroxyphenyl)pyrolidin-2-one and ferulamide [139]. Among all the metabolites tested, only *N*-*trans*-feruloyldopamine and (*E*)-3-(4-hydroxy-3-methoxybenzylidene)-4-(4-hydroxyphenyl) pyrrolidin-2-one showed moderate anti-inflammatory activity on the NO production in lipopolysaccharide (LPS)-induced macrophages’ activities with IC_50_ values of 25.3 ± 0.5 and 23.5 ± 1.7 μM, respectively [139].

NTFT was isolated together with 5 aristolactam alkaloids named dasymaschalolactams A−E (**177**–**181**, Figure 12), dasymaschalolactone (**182**, Figure 12) and 18 other known compounds from the twig extract of *Dasymaschalon dasymaschalum* (Annonaceae). This plant is distributed worldwide in tropical countries in Asia (Thailand and the Malaysian peninsular) and Africa [142]. The known compounds were identified as oldhamactam, velutinam, enterocarpam-III, griffithinam, goniopedalin, taliscanine, duguevalline, desmethoxykanugin, 7,8-dimethoxy-5-hydroxyflavone, alpinetin, 8-hydroxynaringenin-4′-methyl ether, 7-methoxyisobenzofuran-1(3*H*)-one benzyl benzoate, 2-methoxybenzyl benzoate paprazine, (−)-zeylenol and (+)-crotepoxide 4-hydroxybenzaldehyde. NTFT and paprazine showed α-glucosidase inhibition with IC_50_ values of 4.5 and 24.7 μM, respectively [142].

**Figure 13:**
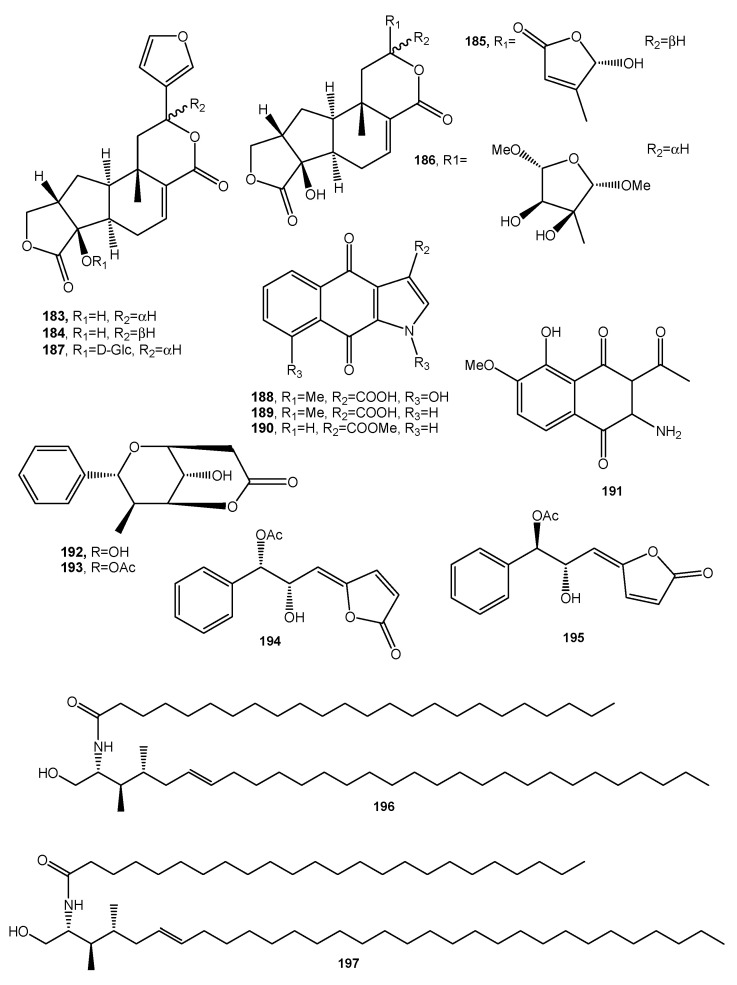
The structures of compounds **183**–**197**.

NTCT, NTFT, five rearranged clerodane diterpenoids, named 4-*epi*-baenzigeride A, its 4-*O*-d-glucoside, 4,12-di-*epi*-baenzigeride A, tinobaenzins A and B (**183, 187, 184**–**1864**, Figure 13), along with four known compounds were isolated from *Tinospora baenzigeri* (Menispermacae) stem organic extract [143]. This plant is widely diffused in Asia, Africa, Australia and the Pacific [143,144,145] and in Thailand its decotion is used in traditional medicine for antipyretic and antimalarial treatment as well as its root extract. The other already known compounds were identified as baenzigeroside B, (+)-lariciresinol, caruilignan D and the aglycone of breyniaionoside D. Only the last two compounds and NTCT showed hepatoprotective activity against *N*-acetyl-*p*-aminophenol (APAP)-induced HepG2 cell damage at 10 μM with 17.0%, 19.2% and 39.0% inhibition, respectively [143].

NTCT, NCCT, NTFT, NCFT and some of their derivatives as well as that of NCAT were identified in fruits, leaves and root barks of *Lycium barbarum* (Solanaceae) by UPLC-Q-Orbitrap-MS/MS [146]. They are widely used in traditional Chinese prescriptions and patent medicines [147,148,149]. The other 131 known compounds were identified using the same method and among them, 98, 28 and 35 constituents were detected in *L. barbarum* fruits, leaves and root barks, respectively. Dicaffeoylspermidine/sperminidine derivatives were the most detected compounds (74/131) while six saponins and 5,6-dihydrosolasonine were reported for the first time in this plant. The root bark extract possessed the strongest antioxidative and cytotoxic activity [146].

NTCT, NTFT, 4 alkaloids named goniochelienic acids A and B, methyl goniochelienate and goniochelieninone (**188**–**191**, Figure 13), 4 styryllactones, named (−)-(4*S*,5*S*,6*R*,7*S*,8*S*)-goniochelienlactone, its 7-*O*-acetyl derivative, (+)-(7*S*,8*S*)-goniochelienbutenolide A and (−)-(7*S*,8*R*)-goniochelienbutenolide B (**192**–**195**, Figure 13), together with 13 known compounds, were isolated from the twig and leaf extracts of *Goniothalamus cheliensis* (Annonaceae) [150]. This large tree is distributed throughout the world, but it is present essentially in southeast Asia [151] and is used in folk medicine to treat fever, scabies, edema, rheumatism, tympanites and typhoid fever [151,152]. The other already known compounds were identified as 3-methyl-1*H*-benz[f]indole-4,9-dione, (−)-goniobutenolide B, 7-*epi*-(−)-goniobutenolide B, (+)-goniodiol, goniodiol-8-monoacetate, (+)-7-*O*-acetylgoniodiol, 8-acetoxy goniofufurone, isoaltholactone, (+)-glaberide I, (−)-glaberide I, (+)-syringaresinol, (+)-medioresinol, (+)-episyringaresinol, (−)-syringaresinol, (−)-episyringaresinol, (−)-pinoresinol, griffithazanone A, cleistopholine, vanillic, *p*-hydroxybenzoic, *p*-methylbenzoic and *trans*-ferulic acids, 4-hydroxy-3-methoxypropiophenon, 3,5-dimethoxy-4-hydroxypropiophenone, *p*-hydroxybenzaldehyde, ethyl-4-hydrozybenzoate, (−)-(3*R*)-mellein methyl ether, derrusnin, 5-hydroxy-7-methoxy-3′,4′-methylene dioxy isoflavone, derrustone, robustone methyl ether, derrugenin, robustigenin and methyl-BRM-5 [150]. Among all the compounds tested for cytotoxicity against human colorectal cancer cells (HCT-116), griffithazanone A was the most potent with an IC_50_ value of 2.39 μM [150].

NTCT was isolated together with 14 alkaloids, including 2 indole alkaloids, 1 quinoline alkaloid, 2 pyridine alkaloids, 4 carbazole alkaloids and 3 amides from the aerial parts of *Clausena lansium* Lour. Skeels (Rutaceae). These metabolites were identified as 3-oxoindole and indole-3-carboxaldehyde, dictamine, murrayanine, claulansine G, clausine I, *O*-demethylmurrayanine, atanine, 4-methoxy-1*H*-quinolin-2-one and 4-methoxy-1-methylquinolin-2-one. Among all the compounds assayed for their cytotoxic activity against Hela cancer cell line, four carbazole alkaloids, murrayanine, claulansine G, clausine I and *O*-demethylmurrayanine, showed weak cytotoxicity with IC_50_ values ranging from 69.31 to 138.32 μM [153].

NTCT was isolated together with speretin, 4-methoxyquinolin-2-one, pinoresinol, medioresinol, syringaresinol, *N*-benzoyl-L-phenylalaninol, L-sesamin, diosmetin, zhebeiresinol, vitexin and isoscopletin from the organic extract of *Zanthoxylum nitidum* (Roxb.) DC. (Rutaceae) leaves. *Z. nitidium* is widely used in traditional Chinese herbal medicines [154].

NTCT, NTCAT and two ceramides, named celtisamides A and B (**196** and **197**, Figure 13) were isolated together with platanic and betulinic acids, the (0.6:0.4) mixture of oleanolic and ursolic acids, friedelin, β-sitosterol, and β-sitosterol 3-*O*-β-d-glucoside and betulinic acid from the stem bark of *Celtis tessmannii* Rendle (Cannabaceae). *p*-Hydroxybenzoyl, *p*-coumaric acid anhydride, glucosyringic acid, *cis*-1-*O*-methyl-inositol and succinic acid were isolated from the root organic extract of the same plant [155]. *C. tessmannii* is used as analgesic and for the treatment of diarrhea, fever, inflammation of respiratory organs, tachycardia, anemia, gangrene, sexual weakness, insomnia, nervosity, muscles pain and malaria [156]. All the metabolites were tested for antiplasmodium and cytotoxic activities. *cis*-1-*O*-Methylinositol (IC_50_ = 14.3 μM) showed the strongest inhibition of urease, while succinic acid (IC_50_ = 12.9 μM) exhibited the best inhibition against lipoxygenase. Succinic acid (IC_50_ = 9.5 μM) showed the best DPPH radical scavenging activity, while betulinic acid exhibited a strong (IC_50_ values ranging from 1.87-2.34 μg/mL) against chloroquine-sensitive (Pf 3D7), and chloroquine-resistant (Pf Dd2 and Pf INDO) strains of *Plasmodium falciparum* [155].

**Figure 14:**
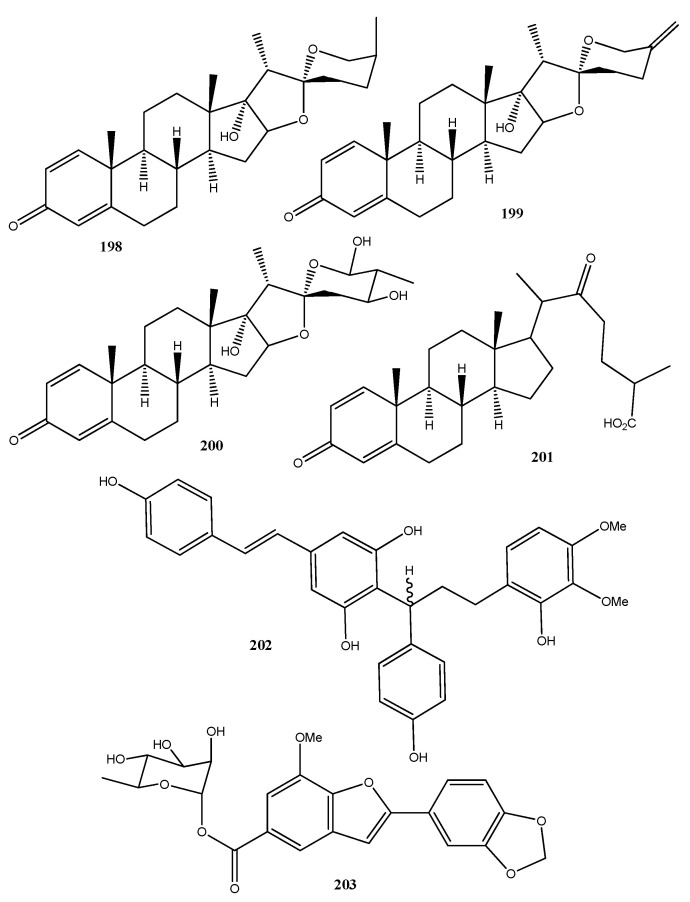
The structures of compounds **198**–**203**.

NTCT, NTFT and NTFO were isolated together with four new steroidal sapogenins, named dracaenogenins C–F (**198**–**201**, Figure 14), a new conjugated chalcone-stilbene, 3′′-methoxycochinchinenene H (**202**, Figure 14), and eight known compounds from the stems of *Dracaena usambarensis* Engl. (Asparagaceae) [157]. The organic extracts of this tree showed anticancer [158], anti-inflammatory [159] and antimicrobial properties [158] and antiestrogenic, antioxidative, and bacteriostatic activities [160]. 3′′-Methoxycochinchinenene H (**202**), 4,4′-dihydroxy-3′-methoxychalcone and grossamide tested at 100 μM were substantially more potent than ibuprofen, inhibiting the release of all the cytokines, IL-1β, IL-2, GM-CSF and TNF-α from 0.06% to 58.04% compared to LPS control. *Trans*-resveratrol significantly reduced the GM-CSF (6.11% of LPS control) and TNF-α (18.35% of LPS control) release [157].

NTCT, NTFT, NTST, a previously undescribed arylbenzofuran rhamnoside named aristolochiaside (**203**, Figure 14) and seven known compounds were isolated from *Isotrema tadungense* (Aristolochiaceae), from which the extract showed significant cytotoxic activity [161]. It is a plant essentially distributed in Vietnam. The already known compounds were identified as aristolactam AIIIa, aristololactam CII, grossamide, cannabisin D, melongenamide, cannabisin F and *N*-*trans*-feruloyldopamine. Among the isolated compounds, aristolochiaside, aristolactam AIIIa and NTST showed strong and selective cytotoxicity on the HeLa human cancer cell line with IC_50_ values of 7.59 ± 1.03, 8.51 ± 1.73 and 9.77 ± 1.25 μM, respectively [161].

**Figure 15:**
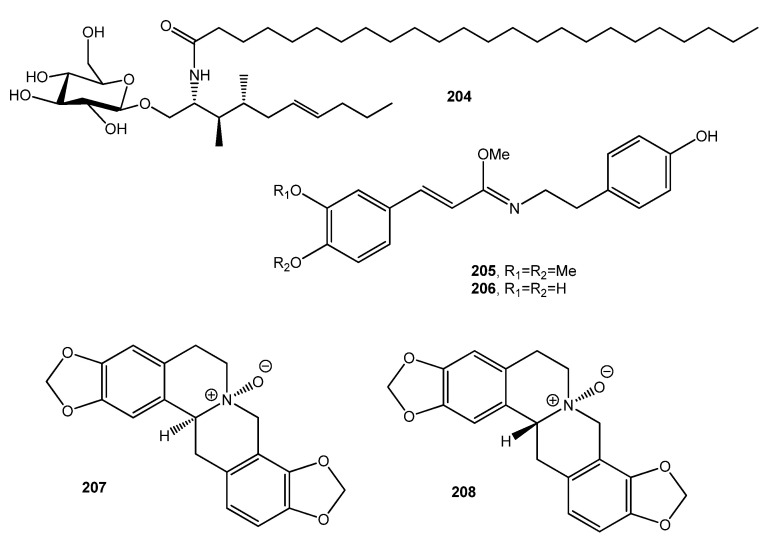
The structures of compounds **204**–**208**.

NTCT, NTFT, NTFO and a previously undescribed cerebroside named eloundemnoside (**204**, Figure 15) were isolated together with 17 known compounds from the roots of *Celtis adolphi*-*friderici* Engl. (Cannabaceae) [162]. This semi-deciduous tree is diffused in the center region of Cameroon and known as “odou” by the Ewondo tribe, where its bark fruits and leaves are used in folk medicine to treat severe cough, fever, headache, tuberculosis and sore eyes [163]. The other known compounds were identified as β-sitosterol, heptacosanoic vanilic azelaic, laceroic hydroxybenzoic and aspartic acids, 3-carboxaldehyde, glycerol, 1-octadecanoate β-sitosterol-3-*O*-β-d-glucopyranoside, sapiol, indole and allantoin [162]. Heptacosanoic, vanilic and azakleic acids showed good antioxidant activities with IC_50_ values of 22.2, 29.3 and 13.2 μM, respectively. Azelaic acid is also a strong inhibitor of lipoxygenase (IC_50_ value of 16.3 μM), while friedelin exhibited the highest inhibition of urease with an IC_50_ value of 15.3 μM. However, all the compounds tested showed a moderate butyrylcholinesterase inhibition [162].

NTFT, NTCAT, NTCT and two previously undescribed phenolic imidates, named fistuloimidates A and B (**205** and **206**, Figure 15), were isolated together with persicoimidate, *N*-coumaroyltyrosine, isorhamnetin-3-*O*-galactopyranoside and 1-*O*-(4-hydroxybenzoyl)-β-d-glucopyranose from the extract of the previously described *A. fistulosum* [164]. Fistuloimidate A (**205**) and 1-*O*-(4-hydroxybenzoyl)-β-d-glucopyranose showed antibacterial activity against *E. coli* with MIC values of 2000 and 1000 μg/mL, respectively, while fistuloimidate B (**206**) showed the same activity against both *E. coli* and *S. aureus* with MIC values of 7.8 and 3.9 μg/mL respectively. Persicoimidate and *N*-coumaroyltyrosine showed the same activity against *S. aureus* with MIC values for both compounds of 250 μg/mL. Among all the compounds tested against the breast cancer cell line MCF-7, persicoimidate and isorhamnetin 3-*O*-galactopyranoside showed low cytotoxic effects in a dose-dependent manner with IC_50_ values of 94.4 ± 5.1 and 94.1 ± 1.8 μg/mL, respectively [164].

NCCT, NTFT, NTCT, 2 previously undescribed tetrahydroprotoberberine, named 7*R*,14*S*-*cis*-tetrahydrocoptisine *N*-oxides and 7*R*,14*R*-*trans*-tetrahydrocoptisine *N*-oxide (**207** and **208**, Figure 15), and 11 known compounds were isolated from the aerial parts of *Chelidonium majus* L. (Papaveraceae) [165]. The known compounds were identified as impatien B, spallidamine, oxychelerythrine, dihydrosanguinarine, *N*-demethyloxysanguinarine, chelidonine, isochelidonine, 4-[formyl-5-methoxymethyl-1*H*-pyrol-1-yl] butanoate, noroxyhydrastinine, 3,4-dehydrotheaspirone and loliolide. 7*R*,14*R*-*trans*-Tetrahydrocoptisine *N*-oxide (**208**), *N*-demethyloxysanguinarine, chelidonine, isochelidonine, NTCT, 4-[formyl-5-methoxymethyl-1*H*-pyrol-1-yl] butanoate and 3,4-dehydrotheaspirone inhibited the nitric oxide production in LPS-induced RAW 264.7 macrophages with the IC_50_ values ranging from 1.1 to 31.9 μM [165].

## 4. Conclusions

The sources and biological activities of both *E*- and *Z*-diastereomers of *p*-coumaroyl-, caffeoyl-, feruloyl-, 5-hydroxyferuloyl- serotonine-, sinapoyl- and tryptamine-tyramine alkylamides and other related alkylamides described in the text are summarized in Table 1, while those of the co-metabolites isolated from the same sources are reported in Table 2. Among the alkylamides, NTCT is that produced by several plants belonging to different species followed by NTFT and NCFT. Some promising activities were also reported for them suggesting their potential use in different fields. However, further studies are needed to determine their mode of actions as well as suitable formulations should be prepared for their practical applications.

## Figures and Tables

**Table 1:** Alkylamides, their sources and biological activities.

Alkylamide	Source	Biological Activity	References
*N*-*trans*-Cinnamoyltyramine (**1**, Scheme 3)	*A. yunnanensis*	No activity	[95]
*N*-*cis*-Feruloyltyramine (NCFT, **2**, Figure 2)	*C. annuum* var. *grossum*	“	[13,14]
	*C. annuum*	“	[29]
	*P. suberosa*	“	[32,34]
	*A. elegans*	“	[38]
	*P. longifolia* var. *pendula*	“	[42]
	*P. hyrcanicum*	“	[82]
	*N. nucifera*	Inhibition of pancreatic lipase	[88]
	*D. cochinchinensis*	No activity	[104]
	*I. batatas*	Inhibition of α-glucosidase	[113]
	*L. chinense*	No activity	[118]
	*N. physaloides*	Inhibition of apoptosis and cytoprotective	[121]
	*L. ruthenicun*	No activity	[129]
	*L. barbarum*	“	[146]
*N*-*trans*-*p*-Coumaroyltyramine (NTCT, paprazine, **4**, Figure 2)	*C. annuum* var. *grossum*	“	[13,14]
	*S. melongena*	“	[15]
	*A. chinense*	Inhibition of thromboxane and prostaglandin synthetase	[23]
	*A. triloba*	No activity	[26]
	*I. maitlandii*	“	[27]
	*C. annuum*	“	[29,30]
	*A. mollissima*	“	[28]
	*P. suberosa*	Anticancer activity and inhibition of protein tyrosine kinases	[32,33,34,35]
	*C. chinensis*	Inhibition of acetylcholinesterase	[36,37]
	*A. elegans*	No activity	[38]
	*P. sanctum*	Antibiotic	[41]
	*P. longifolia* var. *pendula*	No activity	[42]
	*S. tupiniquinorum*	“	[43]
	*P. duclouxii*	“	[44]
	*B. vulgaris*	“	[45]
	*C. asiaticum* var. *sinicum*	“	[52]
	*T. sinensis*	“	[54]
	*P. nigrum*	“	[58]
	*C. gaudichaudianus*	“	[59]
	*D. opposita*	Antidiabetic	[64]
	*A. fistulosum*	No activity	[67]
	*C. annum*	“	[75]
	*S. melongena*	Antidiabetic	[76,103]
	*P. hyrcanicum*	Antiprotozoal	[80]
	*L. chinense*	Moderate radical scavenging, anti-inflammatory and antidiabetic	[83,97,118]
	*P. oleracea*	No activity	[85,135]
	*N. nucifera*	Inhibition of pancreatic lipase	[88]
	*S. buddleifolium*	No activity	[90]
	*C. lansium*	“	[98]
	*Z. mays*	“	[101]
	*H. longipes*	“	[102]
	*D. cochinchinensis*	“	[104]
	*S. cuneata*	“	[105]
	*T. triangulare*	“	[106]
	*D. moniliforme*	“	[110]
	*I. batatas*	Inhibition of α-glucosidase	[113]
	*M. cuneata*	No activity	[114]
	*A. frutescens*	“	[115]
	*C.*-*jobi* var. *mayuen*	“	[119]
	*N. physaloides*	“	[121]
	*A. maurorum*	“	[125]
	*T. terrestris*	“	[127]
	*L. ruthenicun*	“	[129]
	*P. fragrans*	Inhibition of α-glucosidase	[133]
	*A. sagittifolius*	Moderate cytotoxicity	[136]
	*B. retusiusculum*	No activity	[137]
	*F. convolvulus*	“	[138]
	*C. majus*	“	[139]
	*D. dasymaschalum*	Inhibition of α-glucosidase	[142]
	*T. baenzigeri*	Hepatoprotective activity	[143]
	*L. barbarum*	No activity	[146]
	*G. cheliensis*	“	[150]
	*C. lansium*	“	[153]
	*Z. nitidum*	“	[154]
	*C. tessmannii*	“	[155]
	*D. usambarensis*	“	[156]
	*I. tadungense*	“	[161]
	*C. adolphi*-*friderici*	“	[162]
	*A. fistulosum*	“	[164]
	*C. majus*	Inhibition of NO production in RAW 264.7 cells	[165]
*N*-*trans*-Feruloyltyramine (NTFT, **5**, Figure 2)	*C. annuum* var. *grossum*	No activity	[13,14]
	*S. melongena*	“	[15]
	*F. indica*	“	[25]
	*A. triloba*	“	[26]
	*C. annuum*	“	[29]
	*P. suberosa*	“	[32,34]
	*A. elegans*	“	[38]
	*P. sanctum*	“	[41]
	*P. longifolia* var. *pendula*	“	[42]
	*S. tupiniquinorum*	“	[43]
	*P. duclouxii*	“	[44]
	*C. gaudichaudianus*	“	[59]
	*A. fistulosum*	Radical scavenging	[67]
	*S. melongena*	Antidiabetic	[76,103]
	*P. hyrcanicum*	Antiprotozoal	[80]
	*L. chinense*	Moderate radical scavenging, anti-inflammatory and antidiabetic	[83,97]
	*P. oleracea*	Anti-inflammatory	[85,135]
	*N. nucifera*	No activity	[88]
	*S. buddleifolium*	“	[90]
	*P. flaviflorum*	“	[92]
	*Z. mays*	“	[101]
	*D. cochinchinensis*	“	[104]
	*T. triangulare*	“	[106]
	*I. batatas*	Inhibition of α-glucosidase	[113]
	*M. cuneata*	No activity	[114]
	*A. frutescens*	“	[115]
	*N. physaloides*	“	[121]
	*T. terrestris*	“	[127]
	*L. ruthenicun*	“	[129]
	*P. fragrans*	Inhibition of α-glucosidase	[133]
	*F. convolvulus*	No activity	[138]
	*D. dasymaschalum*	Inhibition of α-glucosidase	[142]
	*T. baenzigeri*	No activity	[143]
	*L. barbarum*	“	[146]
	*G. cheliensis*	“	[150]
	*D. usambarensis*	“	[157]
	*I. tadungense*	“	[161]
	*C. adolphi*-*friderici*	“	[162]
	*A. fistulosum*	“	[164]
	*C. majus*	“	[165]
*N*-*trans*-*p*-Coumaroyloctopamine (NTCO, **6**, Figure 2)	*Capsicum annuum* var. *grossum*	“	[13,14]
	*Solanum melongena* L.	“	[15]
	*T. triangulare*	“	[106]
*N*-*trans*-Feruloyloctopamine (NTFO, **7**, Figure 2)	*Capsicum annuum* var. *grossum*	“	[13,14]
	*Solanum melongena* L.	“	[15]
	*D. cochinchinensis*	“	[104]
	*T. triangulare*	“	[106]
	*N. physaloides*	Cytoprotective	[121]
	*L. ruthenicun*	No activity	[129]
	*C. adolphi*-*friderici*	“	[162]
*N*-*cis*-*p*-Cumaroyltyramine (NCCT, **11**, Figure 2)	*A. chinense*	Inhibition of prostaglandin and thromboxane synthetase	[23]
	*A. mollissima*	No activity	[28]
	*C. annuum*	“	[29]
	*A. elegans*	“	[38]
	*P. duclouxii*	“	[44]
	*D. opposita*	“	[64]
	*S. melongena*	Antidiabetic	[76,103]
	*N. nucifera*	No activity	[88]
	*C. lansium*	“	[98]
	*H. longipes*	“	[102]
	*C.*-*jobi* var. *mayuen*	“	[119]
	*L. ruthenicun*	“	[129]
	*L. barbarum*	“	[146]
	*C. majus*	“	[165]
*N*-*trans*-Sinapoyltyramine (NTST, **52**, Figure 4)	*P. longifolia* var. *pendula*	“	[42]
	*I. tadungense*	“	[161]
*N*-*trans*-Caffeoyltyramine (NTCAT, **59**, Figure 5)	*C. asiaticum* var. *sinicum*	“	[52]
	*C. asiaticum*	“	[75]
	*P. hyrcanicum*	Antiprotozoal	[80]
	*L. chinense*	Moderate radical scavengingNF-κB inhibitory	[83,97,118]
	*S. buddleifolium*	No activity	[90]
	*M. cuneata*	“	[114]
	*N. physaloides*	Cytoprotective	[121]
	*F. convolvulus*	No activity	[138]
	*C. tessmannii*	“	[155]
	*A. fistulosum*	“	[164]
3′-Methoxy-NTFT (**86**, Figure 7)	*A. fistulosum*	Radical scavenging	[67]
4′-*O*-Methyl-TNCT (**88**, Figure 7)	*C. annuum*	No activity	[75]
4′-*O*-Methyl-TNCAT (**89**, Figure 7)	*C. annuum*	“	[75]
*N*-*cis*-feruloyloctopamine (NCFO, **91**, Figure 7)	*L. chinense*	Moderate radical scavenging, anti-inflammatory and antidiabetic	[83]
	*L. ruthenicun*	No activity	[129]
	*D. usambarensis*	“	[157]
*N*-*trans*-*p*-oumaroylserotonine (NTCS, **121**, Figure 9)	*Z. mays*	“	[101]
*N*-*trans.*-*p*-coumaroyltryptamine (NTCTR, **122**, Figure 9)	“	“	“
*N*-*trans*-sinapoyloctopamine (NTSO, **123**, Figure 9)	*S. melongena*	“	[103]
*N*-*trans*-caffeoyloctopamine (NTCAO, **124**, Figure 9)	“	“	“
*N*-*trans*-feruloylnoradrenline (NTFA, **125**, Figure 9)	“	“	“
*N*-*cis*-feruloylnoradrenline (NCFA **126**, Figure 9)	“	“	“
*N*-*trans*-*p*-coumaroylnoradrenline (NTCA, **127**, Figure 9)	“	“	“

**Table 2:** Co-metabolites, their sources, and biological activities.

Metabolite	Source	Biological Activity	References
Grossamide (**3**, Figure 2)	*Capsicum annuum* var. *grossum*	No activity	[1,14]
Hordatin A (**8**, Figure 2)	*H. vulgare*	Antifungal	[17]
Hordatin B (**9**, Figure 2)	“	“	“
Hordatin M (**10**, Figure 2)	“	“	“
Lunularic acid (**12**, Figure 2)	*A. chinense*	Inhibition of thromboxane and prostaglandin synthetase	[23]
Rhapontigenin (**13**, Figure 2)	*R. rhabarbarum*	“	[24]
Piceatannol, (**14**, Figure 2)	“	“	“
Rhaponticin (**15**, Figure 2)	“	“	“
Piceatannol glucoside (**16**, Figure 2)	“	“	“
Mandolin S (**17**, Figure 2)	*A. mollissima*	No activity	[29,30]
Mandolin R (**18**, Figure 2)	“	“	“
Mandolin U (**19**, Figure 2)	“	“	“
Mandolin W (**20**, Figure 3)	“	“	“
Mandolin X (**21**, Figure 3)	“	“	“
Canusesnol A (**22**, Figure 3)	*C. annuum*	Cytotoxic	[31]
Canusesnol B (**23**, Figure 3)	“	No activity	“
Canusesnol C (**24**, Figure 3)	“	“	“
Canusesnol D (**25**, Figure 3)	“	“	“
Canusesnol E (**26**, Figure 3)	“	“	“
Canusesnol F (**27**, Figure 3)	“	“	“
Canusesnol G (**28**, Figure 3)	“	“	“
Canusesnol H (**29**, Figure 3)	“	“	“
Canusesnol I (**30**, Figure 3)	“	“	“
Canusesnol J (**31**, Figure 3)	“	“	“
Aristolactam E (**32**, Figure 3)	*A. elegans*	“	[38]
Aristolactam-AIIIa-6-*O*-β-d-glucoside (**33**, Figure 3)	“	“	“
Aristoquinoline A (**34**, Figure 3)	“	“	“
Aristoquinoline B (**35**, Figure 3)	“	“	“
Aristoquinoline C (**36**, Figure 3)	“	“	“
Aristogin F (**37**, Figure 3)	“	“	“
2-Oxo-12-(3′,4′-methylenedioxyphenyl)dodecane (**38**, Figure 4)	*P. sanctum*	Antibiotic	[41]
2-Oxo-14-(3′,4′-methylenedioxyphenyl)tetradecane (**39**, Figure 4)	“	“	“
(**40**, Figure 4)	“	No activity	“
2-Oxo-18-(3′,4′-methylenedioxyphenyl)octadecane (**41**, Figure 4)	“	“	“
2-Oxo-14-(3’,4’-methylenedioxyphenyl)-*trans*-13-tetradecene (**42**, Figure 4)	“	“	“
2-Oxo-16-(3′,4′-methylenedioxyphenyl)-*trans*-15-hexadecene (**43**, Figure 4)	“	Antibiotic	“
2-Oxo-18-(3′,4′-methylenedioxyphenyl)-*trans*-17-octadecene (**44**, Figure 4)	“	No activity	“
2-Oxo-16-phenyl-*trans*-3-hexadecene (**45**, Figure 4)	“	“	“
Methyl [6-(10-phenyldecanyl)tetrahydropyran-2-yl]acetate (**46**, Figure 4)	“	“	“
Methyl 2-(6-tridecyltetrahydro-2*H*-pyran-2-yl)acetate (**47**, Figure 4)	“	“	“
Methyl 2-(5-tetradecyltetrahydro-2-furanyl)acetate (**48**, Figure 4)	“	“	“
2-Oxo-14-(3′,4′-methylenedioxyphenyl)-*trans*-3-tetradecene (**49**, Figure 4)	“	“	“
2-Oxo-16-(3′,4′-methylenedioxyphenyl)-*trans*-3-hexadecene (**50**, Figure 4)	“	“	“
2-Oxo-16-phenyl-3-hexadecane (**51**, Figure 4)	“	“	“
Lignan **53** (Figure 5)	*P. duclouxii*	No activity	[44]
Lignan **54** (Figure 5)	“	“	“
Lignan **55** (Figure 5)	“	Anticancer	“
Lignan **56** (Figure 5)	“	“	“
Lignan **57** (Figure 5)	“	“	“
Lignan **58** (Figure 5)	“	Anti-inflammatory	“
Asiaticumine A (**60**, Figure 5)	*C. asiaticum* var. *sinicum*	No activity	[52]
Asiaticumine B (**61**, Figure 5)	“	“	“
4-Methyl-heptadec-6-enoic acid ethyl ester and (**62** Figure 5)	*T. sinensis*	Antileishmanial	[54]
3-Hydroxy-2,9,11-trimethoxy-5,6-dihydro isoquino[3,2-a] isoquinolinylium (**63** Figure 5)	“	“	“
1-Nitrosoimino-2,4,5-trimethoxybenzene (**64**, Figure 5)	*P. sarmentosum*	Cytotoxic	[58]
Alkaloid **65** (Figure 5)	*C. gaudichaudianus*	No activity	[59]
Alkaloid **66** (Figure 5)	“	“	“
Alkaloid **67** (Figure 5)	“	“	“
Alkaloid **68** (Figure 5)	“	“	“
Eryciboside A (**69**, Figure 6)	*E. hainanesis*	No activity	[60]
Eryciboside B (**70**, Figure 6)	“	Hepatoprotective	“
Eryciboside C (**71**, Figure 6)	“	No activity	“
Eryciboside D (**72**, Figure 6)	“	“	“
Eryciboside E (**73**, Figure 6)	“	“	“
Eryciboside F (**74**, Figure 6)	“	Hepatoprotective	“
Eryciboside G (**75**, Figure 6)	“	No activity	“
Eryciboside H (**76**, Figure 6)	“	“	“
Eryciboside I (**77**, Figure 6)	“	“	“
Eryciboside J (**78**, Figure 6)	“	“	“
Eryciboside K (**79**, Figure 6)	“	“	“
Eryciboside L (**80**, Figure 6)	“	Hepatoprotective	“
Chlorogenic acid derivative (**81**, Figure 6)	“	No activity	“
Chlorogenic acid derivative (**82**, Figure 6)	“	“	“
Chlorogenic acid derivative (**83**, Figure 6)	“	“	“
Chlorogenic acid derivative (**84**, Figure 6)	“	“	“
Biscoumarin (**85**, Figure 6)	“	Hepatoprotective	“
Kaempferol (**87**, Figure 7)	*A. fistulosum*	No activity	[67]
*N*-*trans*-3,4-dimethoxycinnamoyldopamine (**90**, Figure 7)	*P. hyrcanicum*	“	[82]
Neolignanamide (**92**, Figure 7)	*L. chinense*	Moderate radical scavenging	[83]
Neolignanamide (**93**, Figure 7)	“	“	“
Neolignanamide (**94**, Figure 7)	“	“	“
Neolignanamide (**95**, Figure 7)	“	“	“
Neolignanamide (**96**, Figure 7)	“	“	“
Neolignanamide (**97**, Figure 7)	“	“	“
Neolignanamide (**98**, Figure 7)	“	“	“
Lignanamide (**99**, Figure 7)	“	“	“
Portulacaldehyde (**100**, Figure 7)	*P. oleracea*	No activity	[85]
*N*-(*E*)-Feruloyl-4-*O*-methyldopamine (**101**, Figure 8)	“	Anti-inflammatory	“
Nelumnucifoside A (**102**, Figure 8)	*N. nucifera*	No activity	[88]
Nelumnucifoside B (**103**, Figure 8)	“	“	“
Flavifloramide A (**104**, Figure 8)	*P. flaviflorum*	“	[92]
Flavifloramide B (**105**, Figure 8)	“	“	“
Aristoyunnolin I (**106**, Figure 8)	*A. yunnanensis*	“	[95]
Aristoyunnolin I (**107**, Figure 8)	“	“	“
Custonolide (**108**, Figure 8)	“	Moderate cytotoxicity	“
Claulamine C (**109**, Figure 8)	*C. lansium*	No activity	[98]
Claulamine D (**110**, Figure 8)	“	“	“
Claulamine E (**111**, Figure 8)	“	“	“
Clausenaline B (**112**, Figure 8)	“	“	“
Clausenaline C (**113**, Figure 8)	“	“	“
Clausenaline D (**114**, Figure 8)	“	“	“
Clausenaline E (**115**, Figure 9)	“	“	“
Clausenaline F (**116**, Figure 9)	“	“	“
Clausemarins A (**117**, Figure 9)	“	Anti-inflammatory	“
Clausemarin B (**118**, Figure 9)	“	No activity	“
Clausemarin C (**119**, Figure 9)	“	“	“
Clausemarin D (**120**, Figure 9)	“	“	“
(3*R*)-3,7-dihydroxy-8-methoxy-3-(4′-methoxybenzyl)-4-chromanone (**128**, Figure 9)	*D. cochinchinensis*	“	[104]
Sargentodoside A (**129**, Figure 9)	*S. cuneata*	“	[105]
Sargentodoside B (**130**, Figure 9)	“	“	“
Sargentodoside C (**131**, Figure 9)	“	“	“
Sargentodoside D (**132**, Figure 9)	“	“	“
Sargentodoside E (**133**, Figure 9)	“	“	“
Sargentodognan F (**134**, Figure 9)	“	“	“
Sargentodognan G (**135**, Figure 9)	“	“	“
5,6-Dimethoxy-7-hydroxy-8-methyl-flavone (**136**, Figure 9)	*T. triangulare*	“	[106]
5,6-Dimethoxy-8-methyl-2-phenyl-7*H*-1-benzopyran-7-one (**137**, Figure 9)	“	“	“
4-Methoxy-6-(2-hydroxy-4-phenylbutyl)-2*H*-pyran-2-one (**138**, Figure 10)	“	“	“
9,10-Dihydrophenanthrene-1,5-dihydroxy-3,4,7-trimethoxy-9,10-dihydrophenanthrene (**139**, Figure 10)	*D. moniliforme*	“	[110]
Miliusacunine A (**140**, Figure 10)	*M. cuneata*	Antimalaria	[114]
Miliusacunine B (**141**, Figure 10)	“	“	“
Miliusacunine C (**142**, Figure 10)	“	No activity	“
Miliusacunine D (**143**, Figure 10)	“	“	“
Miliusacunine E (**144**, Figure 10)	“	“	“
7-Methoxyflavonol (**145**, Figure 10)	*A. frutescens*	Radical scavenging	[115]
7-Methoxyflavonol (**146**, Figure 10)	“	Inhibition mushroom tyrosinase	“
7-Methoxyflavonol (**147**, Figure 10)	“	“	“
7-Methoxyflavonol (**148**, Figure 10)	“	“	“
7-Methoxyflavonol (**149**, Figure 10)	“	Radical scavenging	“
Fisetinidol glucoside (**150**, Figure 10)	“	“	“
Benzyl glycoside (**151**, Figure 11)	“	“	“
Lyciumsterol A (**152**, Figure 11)	*L. chinense*	No activity	[118]
Lyciumsterol B (**153**, Figure 11)	“	Protective effects on pancreatic islet cells	“
Lyciumsterol C (**154**, Figure 11)	“	“	“
Lyciumsterol D (**155**, Figure 11)	“	No activity	“
Lyciumsterol E (**156**, Figure 11)	“	“	“
Lyciumsterol F (**157**, Figure 11)	“	Protective effects on pancreatic islet cells	“
Lyciumsterol G (**158**, Figure 11)	“	Protective effects on pancreatic islet cells and autophagy activation	“
Lyciumsterol H (**159**, Figure 11)	“	No activity	“
Lyciumsterol I (**160**, Figure 11)	“	Autophagy activation	“
Lyciumsterol J (**161**, Figure 11)	“	No activity	“
Lyciumsterol K (**162**, Figure 10)	“	Autophagy activation	“
(7*R*, 8S)-7-(4-Hydroxy-3,5-dimethoxyphenyl)-8-hydroxy me-thyl-10-[*N*-7″-(4″-hydrxyphenyl)ethyl]carbamoylethenyl-3′-methoxybenzodihydrofuran (**163**, Figure 11)	*N. physaloides*	No activity	[121]
*cis*-*N*-*p*-Hydroxycinnamoyl-7′-methoxyethyltyramine (**164**, Figure 11)	“	“	“
Aristolochic acid II alanine amide (**163**, Figure 11)	*A. maurorum*	“	[125]
*cis*-Terrestriamide (**165**, Figure 11)	*T. terrestris*	“	[127]
Ruthenicunoid A (**166** Figure 11)	*L. ruthenicun*	Inhibition of SIRT1	[129]
Pseuduvarioside (**167**, Figure 12)	*P. fragrans*	No activity	[133]
Oleraisoindole (**168**, Figure 12)	*P. oleracea*	Inhibited NO production in RAW 264.7 cells	[135]
2β,7,3-Trihydroxycalamenene 3-*O*-β-d-glucoside (**170**, Figure 12)	*A. sagittifolius*	Moderate cytotoxicity	[136]
Bobulretulate A (**171** Figure 12)	*B. retusiusculum*	No activity	[137]
Bobulretulate B (**172**, Figure 12)	“	“	“
Majusamide A (**173**, Figure 12)	*C. majus*	“	[139]
Majusamide B (**174**, Figure 12)	“	“	“
Chelidoniumine (**175**, Figure 12)	“	“	“
Tetrahydrocoptisine-*N*-oxide (**176**, Figure 12)	“	“	“
Dasymaschalolactam A (**177**, Figure 12)	*D. dasymaschalum*	“	[142]
Dasymaschalolactam B (**178**, Figure 12)	“	“	“
Dasymaschalolactam C (**179**, Figure 12)	“	“	“
Dasymaschalolactam D (**180**, Figure 12)	“	“	“
Dasymaschalolactam E (**181**, Figure 12)	“	“	“
Dasymaschalolactone (**182**, Figure 12)	“	“	“
4-*epi*-Baenzigeride A (**183**, Figure 13)	*T. baenzigeri*	“	[143]
4,12-di-*epi*-Baenzigeride A (**184**, Figure 13)	“	“	“
Tinobaenzin A (**185**, Figure 13)	“	“	“
Tinobaenzin B (**186**, Figure 13)	“	“	“
4-*O*-d-glucoside (**187**, Figure 13)	“	“	“
Goniochelienic acid A (**188**, Figure 13)	*G. cheliensis*	“	[150]
Goniochelienic acid B (**189**, Figure 13)	“	“	“
Methyl goniochelienate (**190**, Figure 13)	“	“	“
Goniochelieninone (**191**, Figure 13)	“	“	“
(−)-(4*S*,5*S*,6*R*,7*S*,8S)-goniochelienlactone (**192**, Figure 13)	“	“	“
7-*O*-Acetyl derivative of **192** (**193**, Figure 13)	“	“	“
(+)-(7*S*,8*S*)-Goniochelienbutenolide A (**194**, Figure 13)	“	“	“
(−)-(7*S*,8*R*)-Goniochelienbutenolide B (**195**, Figure 13)	“	“	“
Celtisamide A (**196**, Figure 13)	*C. tessmannii*	“	[155]
Celtisamide B (**197**, Figure 13)	“	“	“
Dracaenogenin C (**198**, Figure 14)	*D. usambarensis*	“	[157]
Dracaenogenin D (**199**, Figure 14)	“	“	“
Dracaenogenin E (**200**, Figure 14)	“	“	“
Dracaenogenin F (**201**, Figure 14)	“	“	“
3′′-Methoxycochinchinenene H (**202**, Figure 14)	“	Anti-inflammatory	“
Aristolochiaside (**203**, Figure 14)	*I. tadungense*	Cytotoxic	[161]
Eloundemnoside (**204**, Figure 15)	*C. adolphi-friderici*	Moderate butyrylcholinesterase inhibition	[162]
Fistuloimidate A (**205**, Figure 15)	*A. fistulosum*	Antibiotic	[164]
Fistuloimidate B (**206**, Figure 15)	“	“	“
7*R*,14*S*-*cis*-Tetrahydrocoptisine *N*-oxides and (**207**, Figure 15)	*C. majus*	No activity	[165]
7*R*,14*R*-*trans*-Tetrahydrocoptisine *N*-oxide (**208**, Figure 15)	“	Inhibited NO production in RAW 264.7 cells	“

## Data Availability

Not applicable.
